# Supplementary material for: EV-mediated promotion of myogenic differentiation is dependent on dose, collection medium, and isolation method
Source: Mol Ther Nucleic Acids. 2023 Jul 15;33:511–28. doi: 10.1016/j.omtn.2023.07.005 (PMC10432918; doi:10.1016/j.omtn.2023.07.005)
Supplement: Document S1. Figures S1–S6 and Tables S1–S5 [file mmc1.pdf]

OMTN, Volume *33*

Supplemental information

EV-mediated promotion of myogenic differentiation is dependent on dose, collection medium, and isolation method

Britt Hanson, Ioulia Vorobieva, Wenyi Zheng, Mariana Conceição, Yulia Lomonosova, Imre Mäger, Pier Lorenzo Puri, Samir El Andaloussi, Matthew J.A. Wood, and Thomas C. Roberts

Supplemental Information

Table S1

Target sequences for SMARTpool siRNAs used in this study.

ID	Target sequence (5' to 3')
**siRab27a**	CGGAUGGAGAUUACGAUUA
	CAGGAGAGGUUUCGUAGCU
	GUACAGAGCCAAUGGGCCA
	GGGCAUUGAUUUCAGGGAA
**siRab27b**	GCAGAUUAGAAGCUAGUAU
	GGGAAUAGAUUUUCGGGAA
	GGAAGUCAAUGAACGGCAA
	GCAGAGUAGUCAUAGUGUU
**siCtrl**	UGGUUUACAUGUCGACUAA
	UGGUUUACAUGUUGUGUGA
	UGGUUUACAUGUUUUCUGA
	UGGUUUACAUGUUUUCCUA

Table S2

Antibodies used in this study.

MF 20 primary monoclonal antibody, developed by Fischman, D.A. at Weill Cornell Medical College, was obtained from the Developmental Studies Hybridoma Bank, created by the NICHD of the NIH and maintained at The University of Iowa, Department of Biology, Iowa City, IA 52242, USA. WB, western blot, IF, immunofluorescence.

Target protein	Host	Product ID	Manufacturer	Dilution
**Western Blot**				
**Primary Abs**				
Anti-PDCD6IP (Alix)	Mouse	ab117600	Abcam	1:1,000
Anti-CANX (Calnexin)	Rabbit	ab22595	Abcam	1:1,000
Anti-CD9	Rabbit	ab92726	Abcam	1:2,000
**Secondary Abs**				
Anti-mouse IgG, HRP-linked	Horse	7076	Cell Signaling Technology	1:5,000
**Immunofluorescence**				
**Primary Abs**				
Anti-MHC	Mouse	MF 20	Abcam	1:20
**Secondary Abs**				
Anti-rat IgG Alexa Fluor 488	Goat	ab150157	Abcam	1:500

Table S3

RT-qPCR primer sequences used in this study.

ID	Sequence (5' to 3')
qRab27a-Fwd	CGACCTGACAAATGAGCAAAG
qRab27a-Rev	CCTCTTTCACTGCCCTCTG
qRab27b-Fwd	CAGACCTGCCAGACCAAAG
qRab27b-Rev	AGCGTTTCCACTGACTTCTC
qRplp0-Fwd	AAGCAAAGGAAGAGTCGGAG
qRplp0-Rev	CCAGACCGGAGTTTTAAGAGAAG

Table S4

miRNA small RNA TaqMan RT-qPCR assays used in this study.

All products were purchased from Thermo Fisher Scientific.

Target	Product ID	Detection Channel
**mmu-miR-1a-3p**	002222	FAM
**mmu-miR-133a-3p**	002246	FAM
**mmu-miR-206-3p**	000510	FAM
**cel-miR-39**	000200	FAM

Table S5

List of oligonucleotides used in this study.

Oligonucleotides were purchased from IDT (Leuven, Belgium).

ID	Sequence (5' to 3')
**mmu-miR-1a-3p**	UGGAAUGUAAAGAAGUAUGUAU
**mmu-miR-133a-3p**	UUUGGUCCCCUUCAACCAGCUG
**mmu-miR-206-3p**	UGGAAUGUAAGGAAGUGUGUGG
**cel-miR-39**	UCACCGGGUGUAAAUCAGCUUG

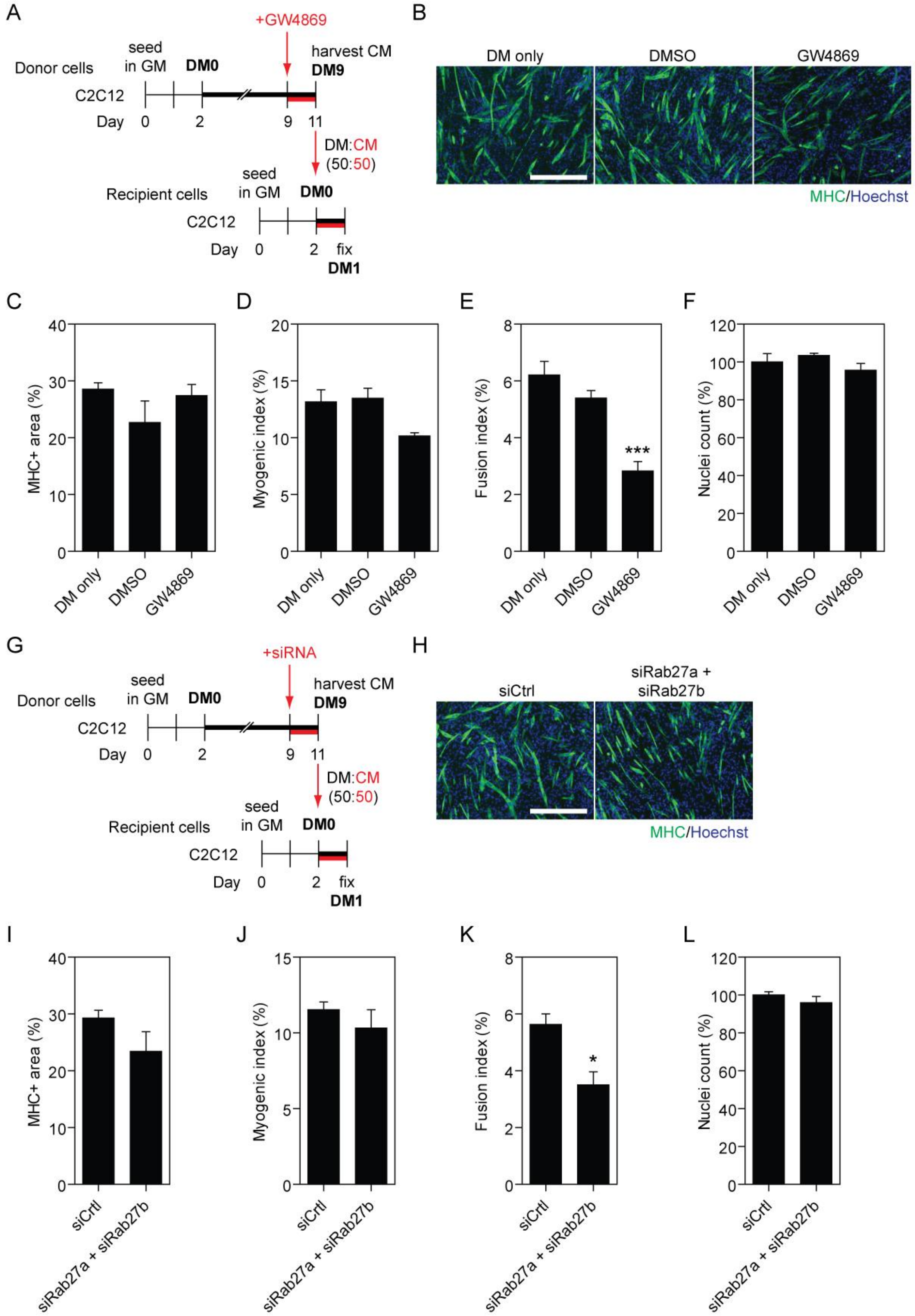
A
+GW4869
Donor cells
seed in GM
DM0
harvest CM
DM9
C2C12
Day 0 2 9 11
DM:CM (50:50)
Recipient cells
seed in GM
DM0
C2C12
Day 0 2 fix
DM1
B
DM only
DMSO
GW4869
MHC/Hoechst
C
MHC+ area (%)
D
Myogenic index (%)
E
Fusion index (%)
***
F
Nuclei count (%)
DM only
DMSO
GW4869
G
+siRNA
Donor cells
seed in GM
DM0
harvest CM
DM9
C2C12
Day 0 2 9 11
DM:CM (50:50)
Recipient cells
seed in GM
DM0
C2C12
Day 0 2 fix
DM1
H
siCtrl
siRab27a + siRab27b
MHC/Hoechst
I
MHC+ area (%)
J
Myogenic index (%)
K
Fusion index (%)
*
L
Nuclei count (%)
siCrtl
siRab27a + siRab27b

Figure S1

Treatment of donor cultures with exosome biogenesis inhibitors reduces myogenic differentiation in recipient cultures following conditioned media transfer.

(**A**) Donor C2C12 cells were cultured in GM for two days and then switched to DM for seven days. Cultures were treated with 10 µM GW4869 and conditioned media (CM) collected 2 days later. Untreated (DM only) and DMSO treated cultures served as controls. Recipient cultures were treated with a 50:50 mixture of CM and fresh differentiation media (DM) at the point of initiating differentiation. Cells were fixed after 24 hours and myogenic differentiation was assessed in the recipient cultures by (**B**) MHC IF, and quantified by (**C**) measuring the MHC+ area, and by calculating the (**D**) myogenic and (**E**) fusion indices. (**F**) The total number of nuclei per representative field of view are shown as a percentage relative to the control group. (**G**) Donor C2C12 cells were cultured as above, and treated with a 50:50 mixture of siRNAs targeting the exosome biogenesis factors *Rab27a* and *Rab27b* (final concentration 100 nM) at differentiation day 7. Conditioned media was harvested 2 days later. A non-targeting siRNA (siCtrl) served as a control). Recipient cultures were treated with a 50:50 mixture of CM and fresh differentiation media (DM) at the point of initiating differentiation. Cells were fixed after 24 hours and myogenic differentiation was assessed in the recipient cultures by (**H**) MHC IF, and quantified by (**I**) measuring the MHC+ area, and by calculating the (**J**) myogenic and (K) fusion indices. (**L**) The total number of nuclei per representative field of view are shown as a percentage relative to the control group. All microscopy images were taken at 10× magnification. Scale bar represents 400 µm. Values are mean + SEM (n=4). Statistical significance was determined by one-way ANOVA with Bonferroni *post hoc* test or Student's t-test, as appropriate, ***P<0.001, *P<0.05.

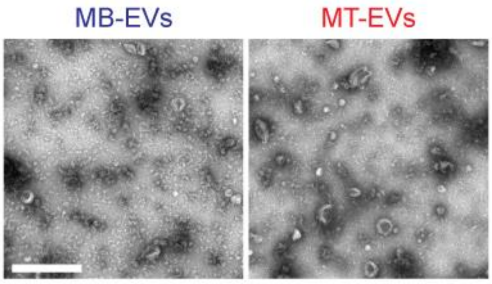

Figure S2

Analysis of EV preparations by transmission electron microscopy.

EVs were collected from myoblasts (MBs) or myotubes (MTs) as described in **Figure 3A** and preparations were analysed by transmission electron microscopy. Vesicles with 'cup-shaped' morphology characteristic of exosomes are observed. Scale bars represent 500 nm.

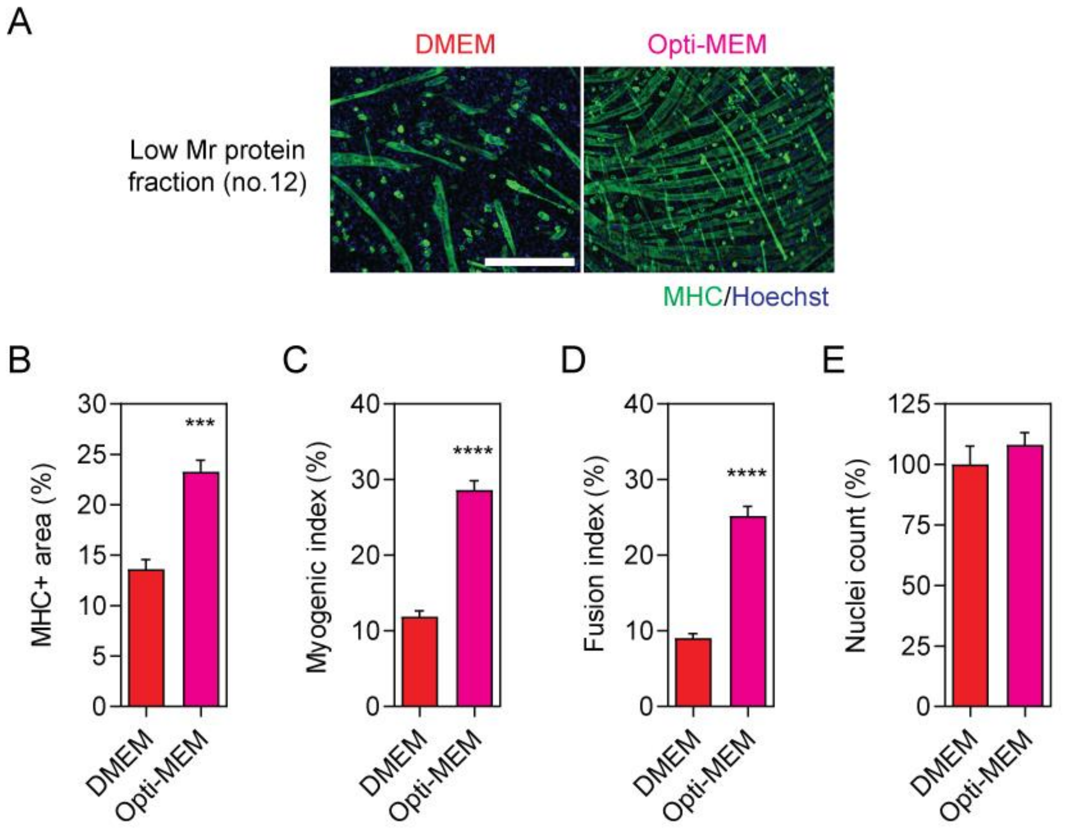

Figure S3

Low molecular weight protein obtained from unconditioned Opti-MEM media is highly pro-myogenic.

Fresh Opti-MEM and DMEM were fractionated by UF-SEC and protein fraction no. 12 collected as shown in **Figure 5B**. Recipient C2C12 cultures were treated with 1 µg/ml of purified protein fraction at the time of switching to DM. (**A**) Myogenic differentiation was assessed by MHC IF at DM2 and quantified by measuring (**B**) MHC+ area, (**C**) myogenic index, and (**D**) fusion index. (**E**) The total number of nuclei per representative field of view are shown as a percentage relative to the control group. All microscopy images were taken at 10× magnification. Scale bar represents 400 µm. All values are mean + SEM (n=4). Statistical significance was determined by a Student's t test, ***P<0.001, ****P<0.0001.

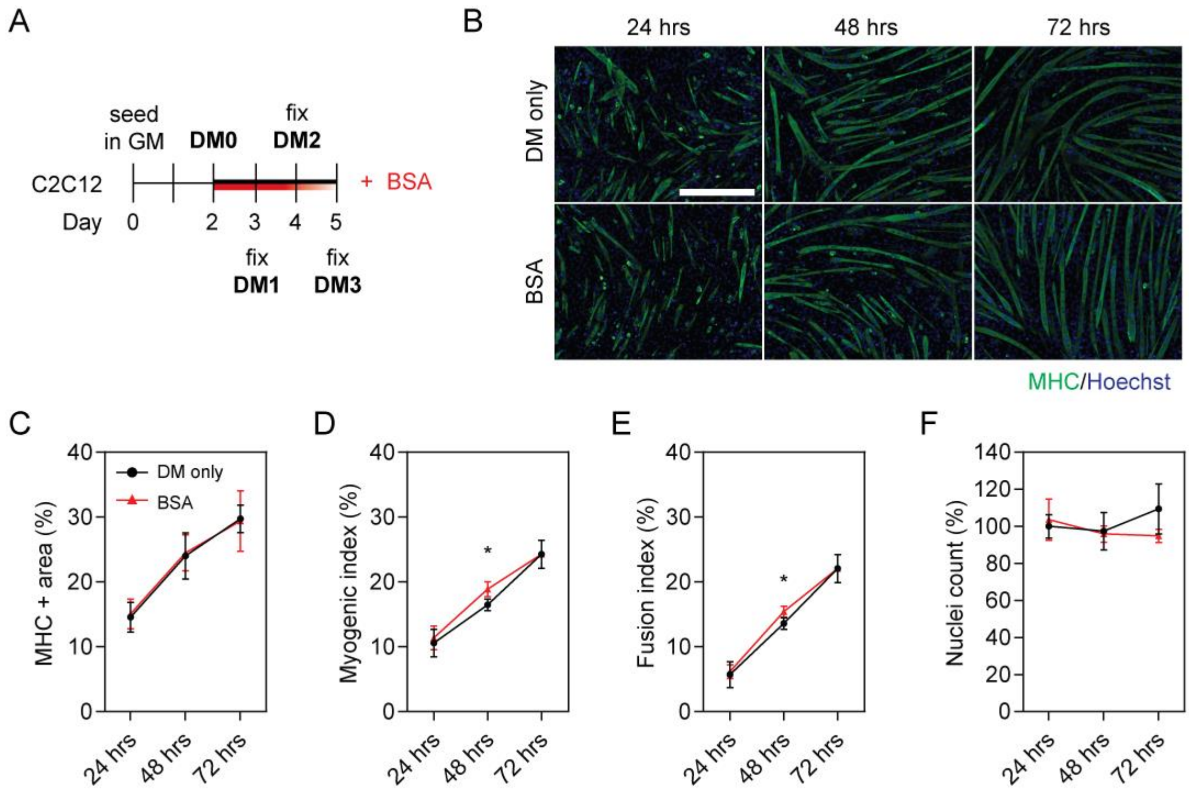

Figure S4

A non-specific increase in extracellular protein does not enhance myogenic differentiation.

(**A**) C2C12 myoblasts were grown in GM for two days, and treated with 5 µg/ml of bovine serum albumin (BSA) at the time of switching to DM. (**B**) Myogenic differentiation was assessed by MHC IF at DM1, DM2 and DM3, and quantified by measuring (**C**) MHC+ area, (**D**) myogenic index, and (**E**) fusion index. Untreated (DM only) cultures were included as negative controls. (**F**) The total number of nuclei per representative field of view are shown as a percentage relative to the control group. All microscopy images were taken at 10× magnification. Scale bar represents 400 µm. All values are mean ± SEM (n=4). Statistical significance was determined by Student's t test, *P<0.05.

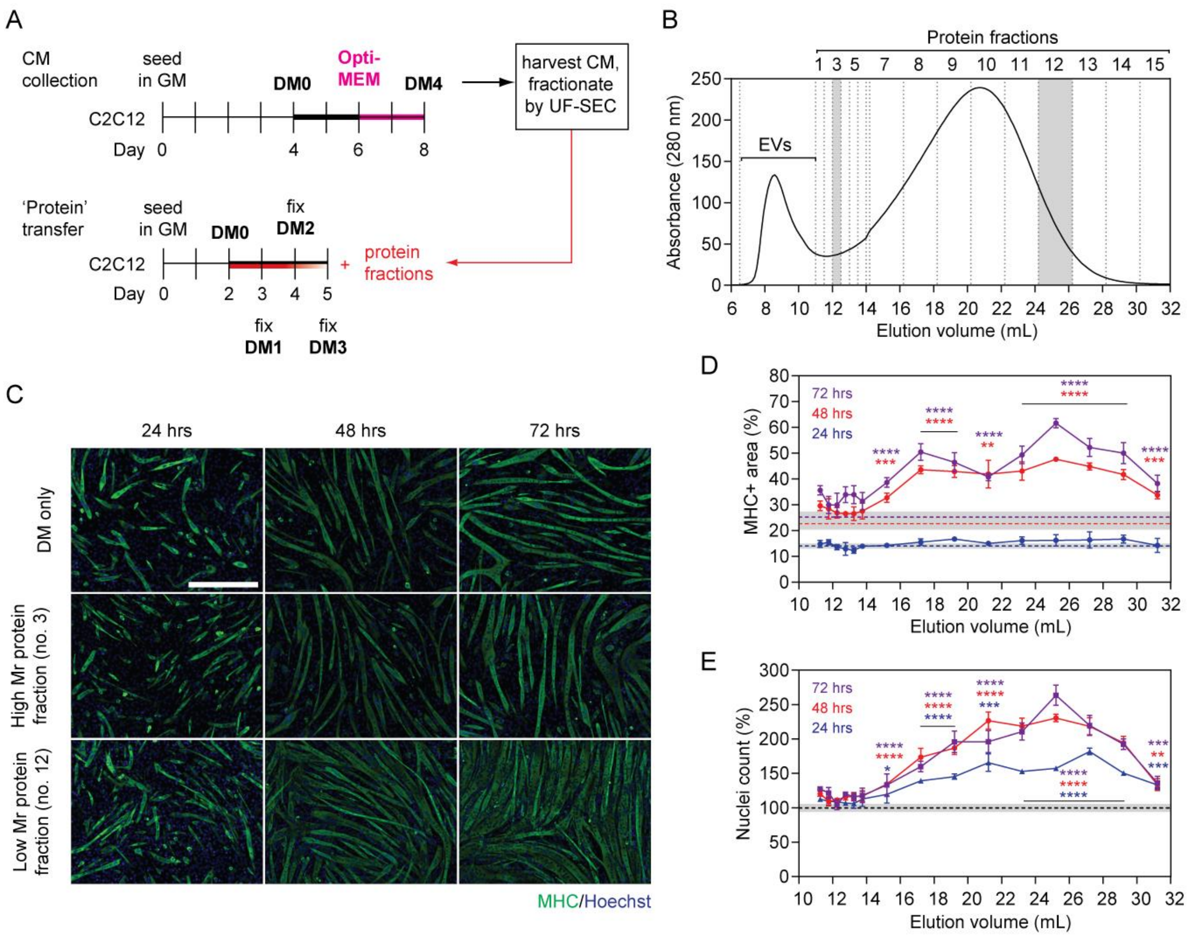
A
CM collection
seed in GM
Opti-MEM
DM0
DM4
C2C12
Day 0 4 6 8
harvest CM, fractionate by UF-SEC
'Protein' transfer
seed in GM
DM0
fix DM2
C2C12
Day 0 2 3 4 5
+ protein fractions
fix DM1
fix DM3
B
Protein fractions
1 3 5 7 8 9 10 11 12 13 14 15
EVs
Absorbance (280 nm)
Elution volume (mL)
C
24 hrs
48 hrs
72 hrs
DM only
High Mr protein fraction (no. 3)
Low Mr protein fraction (no. 12)
MHC/Hoechst
D
72 hrs
48 hrs
24 hrs
MHC+ area (%)
Elution volume (mL)
E
72 hrs
48 hrs
24 hrs
Nuclei count (%)
Elution volume (mL)

Figure S5

Low molecular weight myotube-derived secreted protein enhances myogenic differentiation when purified from Opti-MEM collection media.

(**A**) C2C12 myotube-derived secreted protein fractions were isolated by UF-SEC from CM collected in Opti-MEM. Recipient cultures were treated with 1 μg/ml of purified extracellular protein from each fraction at the time of switching to DM. (**B**) The non-vesicular-associated protein eluates from the SEC column were measured by UV spectrophotometry absorbance at 280 nm. Two fractions (no. 3 and no. 12) were selected to represent high and low Mr extracellular protein fractions, respectively (indicated by grey shading). (**C**) Myogenic differentiation in recipient cultures was assessed by MHC IF at DM1 (24 hours), DM2 (48 hours), and DM3 (72 hours). Representative images from the high and low Mr extracellular protein fractions are shown from each time point. (**D**) Myogenic differentiation was quantified by measuring the MHC+ area. The means of corresponding DM only control groups from each time point are shown ± SEM (grey shaded area). (**E**) The total number of nuclei per representative field of view are shown as a percentage relative to the control group (data were scaled such that the mean of the control group was returned to a value of 100%). Untreated (DM only) cultures were included as negative controls. All microscopy images were taken at 10× magnification. Scale bar represents 400 μm. Values are mean ± SEM (n=4). Statistical significance was determined by a Student's t-test, **P<0.01, ***P<0.001, and ****P<0.0001 (for ease of interpretation test results are only shown for fraction nos. 7-15). The colour of the significance indicators corresponds to the respective comparison relative to the DM only control at each time point (i.e. blue for the 24 hour time point, red for 48 hours, and purple for 72 hours).

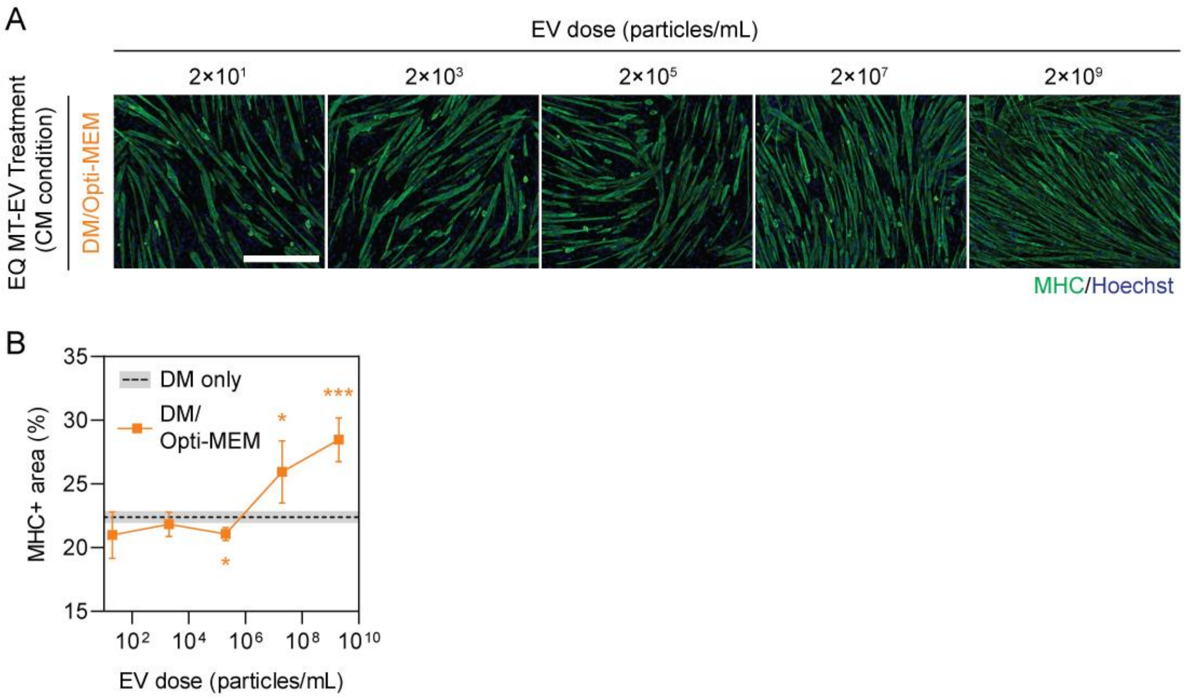
A
EV dose (particles/mL)
2×10¹
2×10³
2×10⁵
2×10⁷
2×10⁹
EQ MT-EV Treatment
(CM condition)
DM/Opti-MEM
MHC/Hoechst
B
MHC+ area (%)
DM only
DM/
Opti-MEM
EV dose (particles/mL)

Figure S6

EQ MT-EVs obtained from Opti-MEM collection media exhibit pro-myogenic effects at high doses.

EVs were isolated by ExoQuick polymer precipitation (EQ MT-EVs) from C2C12 myotubes and collected in Opti-MEM isolation media. Myoblasts were grown in GM for four days, DM for two days, and isolation medium (Opti-MEM) for a further two days. Recipient myoblast cultures were treated with a range of EQ MT-EV doses. (**A**) Myogenic differentiation was assessed by MHC IF at DM2 and quantified by (**B**) measuring the MHC+ area. All microscopy images were taken at 10× magnification. Scale bar represents 400 µm. All values are mean ± SEM (*n*=4). Untreated (DM only) cultures were included as negative controls. Statistical significance was determined by Student's *t*-test, *$P<0.05$, ***$P<0.001$.
