# Supplementary material for: EV-mediated promotion of myogenic differentiation is dependent on dose, collection medium, and isolation method
Source: Mol Ther Nucleic Acids. 2023 Jul 15;33:511–28. doi: 10.1016/j.omtn.2023.07.005 (PMC10432918; doi:10.1016/j.omtn.2023.07.005)
Supplement: Document S2. Article plus supplemental information [file mmc2.pdf]

Molecular Therapy
Nucleic Acids
Original Article

EV-mediated promotion of myogenic differentiation is dependent on dose, collection medium, and isolation method

Britt Hanson,[1,2] Ioulia Vorobieva,[1,3] Wenyi Zheng,[4] Mariana Conceição,[1,3] Yulia Lomonosova,[1,3] Imre Mäger,[1,2] Pier Lorenzo Puri,[5] Samir El Andaloussi,[4] Matthew J.A. Wood,[1,2,3,6] and Thomas C. Roberts[1,2,3,6]

[1]Department of Paediatrics, University of Oxford, South Parks Road, Oxford OX1 3QX, UK; [2]Department of Physiology, Anatomy and Genetics, University of Oxford, South Parks Road, Oxford OX1 3QX, UK; [3]Institute of Developmental and Regenerative Medicine, University of Oxford, IMS-Tetsuya Nakamura Building, Old Road Campus, Roosevelt Dr, Headington, Oxford OX3 7TY, UK; [4]Department of Laboratory Medicine, Karolinska Institutet, Huddinge SE-141 86, Sweden; [5]Sanford Burnham Prebys Medical Discovery Institute, Development, Aging and Regeneration Program, La Jolla, CA 92037, USA; [6]MDUK Oxford Neuromuscular Centre, South Parks Road, Oxford OX3 7TY, UK

Extracellular vesicles (EVs) have been implicated in the regulation of myogenic differentiation. C2C12 murine myoblast differentiation was reduced following treatment with GW4869 or heparin (to inhibit exosome biogenesis and EV uptake, respectively). Conversely, treatment with C2C12 myotube-conditioned medium enhanced myogenic differentiation. Ultrafiltration-size exclusion liquid chromatography (UF-SEC) was used to isolate EVs and non-EV extracellular protein in parallel from C2C12 myoblast- and myotube-conditioned medium. UF-SEC-purified EVs promoted myogenic differentiation at low doses (≤2 × 10^8 particles/mL) and were inhibitory at the highest dose tested (2 × 10^{11} particles/mL). Conversely, extracellular protein fractions had no effect on myogenic differentiation. While the transfer of muscle-enriched miRNAs (myomiRs) has been proposed to mediate the pro-myogenic effects of EVs, we observed that they are scarce in EVs (e.g., 1 copy of miR-133a-3p per 195 EVs). Furthermore, we observed pro-myogenic effects with undifferentiated myoblast-derived EVs, in which myomiR concentrations are even lower, suggestive of a myomiR-independent mechanism underlying the observed pro-myogenic effects. During these investigations we identified technical factors with profound confounding effects on myogenic differentiation. Specifically, co-purification of insulin (a component of Opti-MEM) in non-EV LC fractions and polymer precipitated EV preparations. These findings provide further evidence that polymer-based precipitation techniques should be avoided in EV research.

INTRODUCTION

Extracellular vesicles (EVs) are nano-sized vesicles secreted by the majority of cells, and which are involved in cell-to-cell communication via the transfer of biological macromolecules (e.g., DNA, RNA, and protein).[1] Of particular interest are extracellular microRNAs (ex-miRNAs) which have been proposed to act as paracrine signaling factors. The myomiRs (miR-1, miR-133a, and miR-206) are a set of miRNAs that are highly enriched in skeletal muscle, and are involved in the regulation of myogenic differentiation.[2–4] Serum myomiRs have been proposed as minimally invasive biomarkers in the context of multiple inherited and acquired muscle pathologies.[5] For example, myomiRs are highly elevated in the serum of Duchenne muscular dystrophy patients and dystrophic animal models.[6–12] Release of ex-myomiRs is subject to a degree of selectivity, and is associated with muscle turnover, periods of muscle regeneration, and myogenic differentiation of myoblasts in culture.[5,6,9,13] MyomiRs are detectable in muscle-derived EVs,[6,9,14] although the majority (~99%) of ex-myomiRs are non-vesicular, and are instead most likely protected from exonucleolytic degradation through the formation of protein or lipoprotein complexes.[6,9]

We have proposed a model whereby mature muscle releases EV-encapsulated myomiRs, which are taken up by immature cells in muscle (e.g., satellite cells), to promote their activation, and thereby support muscle growth and repair.[5] Consistent with this hypothesis, multiple studies have reported the cell-to-cell functional transfer of pro-myogenic factors, which could include myomiRs, between donor and recipient C2C12 murine myoblast cells,[15,16] C2C12 myotubes and neuronal cells (NSC-34),[17] activated myogenic progenitor cells (satellite cells) and myofibroblasts,[18] human skeletal myoblasts and human adipose-derived stem cells,[19] and human mesenchymal stem cells (MSCs) and mouse myofibers *in vivo*.[20] The transfer of EVs resulted in enhanced myogenic differentiation in recipient cell cultures[15,19,21] and improved wound repair *in vivo*.[20] Importantly, these studies have utilized methods of EV isolation that are known to co-purify other non-vesicular proteins and soluble factors, such

Received 11 December 2022; accepted 11 July 2023;
https://doi.org/10.1016/j.omtn.2023.07.005.

Correspondence: Thomas C. Roberts, Institute of Developmental and Regenerative Medicine, University of Oxford, IMS-Tetsuya Nakamura Building, Old Road Campus, Roosevelt Dr, Headington, Oxford OX3 7TY, UK.
E-mail: thomas.roberts@idrm.ox.ac.uk

This is an open access article under the CC BY license (http://creativecommons.org/licenses/by/4.0/).

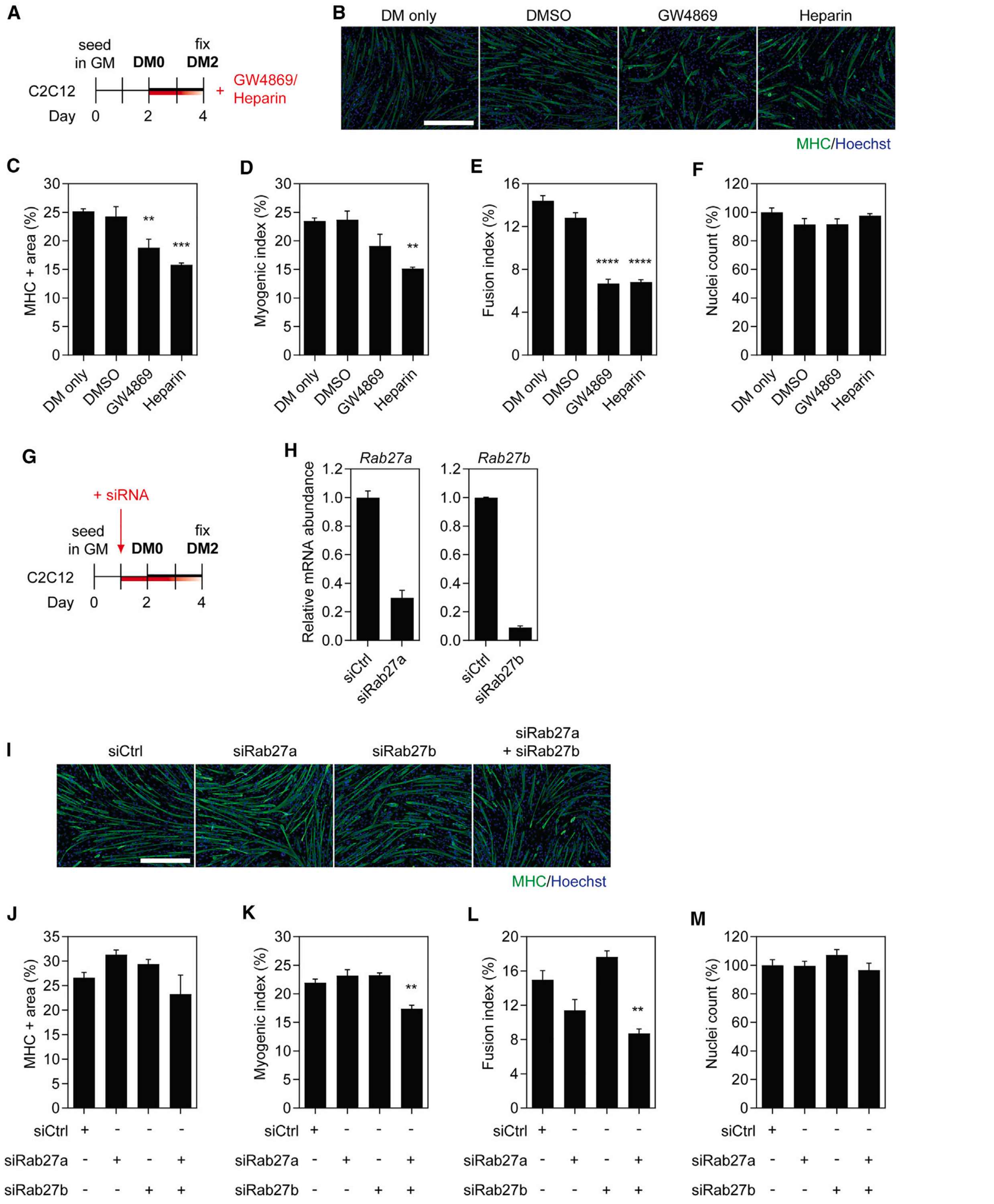

Figure 1. Inhibition of EV release and uptake impairs myoblast differentiation

(A) C2C12 cells were cultured in GM for 2 days and then switched to DM for a further 2 days. Cultures were treated with 10 μM GW4869 (exosome biogenesis inhibitor) or 10 μg/mL heparin (EV uptake inhibitor) at the time of switching to DM. Untreated (DM only) and DMSO-treated cultures were included as negative controls. (B) Myogenic differentiation was assessed by immunofluorescence (IF) staining for myosin heavy chain (MHC), and quantified by calculating the (C) MHC+ area, (D) myogenic index, and

(legend continued on next page)

as ultracentrifugation[19,21] and commercial exosome isolation kits,[15,20] which could have confounded the pro-myogenic effects observed.

While cell-to-cell transfer of EV-associated miRNAs has been widely reported, the biological significance of non-vesicular ex-miRNAs, if any, is less clear. Notably, transfer of high-density lipoprotein-associated myomiRs was found to be ineffective in an *in vitro* model of cardiovascular disease.[22]

In this study we aimed to determine whether C2C12-derived EVs and non-EV soluble protein fractions can stimulate myogenic differentiation in recipient C2C12 cultures. Here, we have investigated possible roles of vesicular and non-vesicular paracrine signaling in myoblast cultures using high purity methods of EV isolation. We show that EVs can promote myogenic differentiation, although the effect sizes are generally small and opposite phenotypic outcomes are observed depending on the dose used. Conversely, extracellular protein prepared in parallel with EVs did not affect myogenic differentiation. This effect was also shown to be miRNA independent.

During the course of our investigations we identified two technical issues that constitute potential pitfalls for research into myogenic EVs (with relevance in other contexts). Specifically, if Opti-MEM is used as an isolation medium, pro-myogenic effects are observed for non-EV fractions, indicative of contamination with a pro-myogenic soluble protein. (Notably, insulin is a major protein component of Opti-MEM.)

Similarly, the use of polymer precipitation to isolate EVs also stimulated myogenic differentiation in an Opti-MEM-dependent manner. This study has identified Opti-MEM as a potential confounding factor in EV transfer experiments and adds to a growing body of evidence that polymer precipitation techniques should be avoided in EV research.

RESULTS

Inhibition of exosome biogenesis and EV uptake suppresses myoblast differentiation

C2C12 murine myoblasts (MBs) undergo differentiation upon serum withdrawal and fuse to form multinucleate myotubes (MTs), recapitulating the process of *in vivo* muscle regeneration. The secretome is altered during differentiation,[23,24] such that the factors released by differentiating C2C12 myotubes condition the culture medium with a heterogeneous pool of biomolecules. To investigate whether EV-mediated cell-to-cell communication contributes to myogenic differentiation, C2C12 cells cultured in pro-differentiation conditions were treated with inhibitors of exosome biogenesis (i.e., GW4869, an inhibitor of neutral sphingomyelinase 2)[25] and EV uptake (i.e., heparin).[26] C2C12 cells were treated with the inhibitors at the time of switching to differentiation medium (DM) (Figure 1A) and myogenic differentiation assessed by immunofluorescence (IF) staining for myosin heavy chain (MHC) 2 days later (Figure 1B). Untreated (DM only) and dimethyl sulfoxide (DMSO)-treated cultures were included as negative controls. GW4869 treatment inhibited myogenic differentiation, resulting in a ~25% ($p < 0.01$) and ~50% ($p < 0.0001$) reduction in the MHC+ area and fusion index relative to the untreated control group, respectively (Figures 1C and 1E). The myogenic index was reduced by ~18%, although this effect did not reach statistical significance at the $p < 0.05$ level (Figure 1D). The effect of heparin treatment similarly resulted in a reduction in the MHC+ area, in addition to the myogenic and fusion indices, by ~35% ($p < 0.01$), ~35% ($p < 0.01$), and ~50% ($p < 0.0001$) relative to the untreated control group, respectively (Figures 1C–1E). In contrast, C2C12 myocyte proliferation was unaffected by either treatment (Figure 1F). Furthermore, treatment with DMSO had no effect on either myogenic differentiation or cell proliferation (Figures 1B–1F). These results demonstrate that small molecule-mediated inhibition of exosome release and EV uptake impairs myogenic differentiation and suppresses MT maturation. These data support the notion that C2C12 MT-derived EVs play an important pro-myogenic role through paracrine signaling during myogenic differentiation.

To assess the involvement of the specific genes associated with exosome biogenesis on myogenic differentiation, we next inhibited expression of the Rab GTPases RAB27A and RAB27B[27] by RNA interference. RAB27A and RAB27B have been found to play a role in the docking of multivesicular bodies at the plasma membrane, and knocking down these Rab GTPases decreased exosome secretion without resulting in major changes in the secretion of soluble proteins.[27] C2C12 cells were treated with short interfering RNAs (siRNAs) targeting the *Rab27a* and *Rab27b* transcripts (either separately or in combination) 1 day before switching to DM (Figure 1G). Transcript levels of *Rab27a* and *Rab27b* were reduced by ~70% and ~90%, respectively, relative to the non-targeting control (Figure 1H). The extent of C2C12 myogenic differentiation was assessed 2 days later by MHC IF (Figure 1I). There was no significant effect on the overall MHC+ area between the treatments relative to the control (Figure 1J). No difference in myogenic or fusion indices was observed when cultures were treated with siRNAs against either *Rab27a* or *Rab27b* alone. However, a significant >20% reduction in myogenic

(E) fusion index. (F) The total number of nuclei per representative field of view are shown as a percentage relative to the control group. (G) C2C12 cells were cultured in GM for 2 days and then switched to DM for 2 days. Cultures were treated with siRNAs targeted to two exosome biogenesis factors, *Rab27a* and *Rab27b*, separately or in combination, at a final concentration of 100 nM one day before switching to DM. Treatment with a non-targeting siRNA was included as a negative control. (H) *Rab27a* and *Rab27b* mRNA levels were determined by RT-qPCR, normalized to the *Rplp0* reference gene. The data were scaled such that the mean of the control group was returned to a value of 1 (n = 2). (I) Myogenic differentiation was assessed by MHC IF, and quantified by (J) measuring the MHC+ area, and by calculating the (K) myogenic and (L) fusion indices. (M) The total number of nuclei per representative field of view are shown as a percentage relative to the control group. Cultures treated with a non-targeting siRNA pool were included as negative controls. All microscopy images were taken at 10× magnification. Scale bars, 400 μm. Values are mean + SEM (n = 4). Statistical significance was determined by one-way ANOVA with Bonferroni post hoc test, **$p < 0.01$.

index ($p < 0.01$) (Figure 1K) and >40% reduction in the fusion index ($p < 0.01$) (Figure 1L) was observed for the combination treatment where both transcripts were knocked down simultaneously. No changes in the number of nuclei were observed for any of the siRNA treatment groups (Figure 1M). These results suggest that RAB27A and RAB27B are both required for the production of pro-myogenic exosomes, but that the functions of these Rab GTPases may be redundant to some extent. The level of inhibition of MT fusion with knockdown of both *Rab27a* and *Rab27b* was similar to that achieved with GW4869 or heparin treatment (Figures 1A–1F).

Inhibition of exosome biogenesis via either treatment with GW4869 or the combination of siRNAs targeting *Rab27a* and *Rab27b* in donor cultures reduced myogenic differentiation in recipient cultures following conditioned media transfer, which manifested as a statistically significant reduction in fusion index for both treatments (Figure S1).

Transfer of myotube-conditioned medium and ultrafiltration-purified EVs enhances myogenic differentiation

We next sought to further investigate whether pro-myogenic paracrine factors secreted by C2C12 myotubes could enhance myogenic differentiation when transferred between cell culture dishes. C2C12 myotube-conditioned differentiation medium (MT-CM) was harvested from cultures in the late stages of differentiation (i.e., at DM7) and then subjected to sequential ultrafiltration (UF) using 300, 100, and 10 kDa molecular weight (Mr) cutoff filters. The resulting four fractions obtained from this process therefore consisted of (1) crude unfractionated CM, (2) CM containing EVs and other particles >300 kDa in size, and EV-depleted CM containing soluble proteins with an Mr of (3) 100–300 kDa or (4) 10–100 kDa (Figure 2A). Each of these fractions was mixed with an equal volume (50:50) of fresh DM and transferred to C2C12 myoblasts at DM0 (Figure 2A). The effect of treatment with each fraction on myogenic differentiation (by MHC IF) and cell proliferation (by EdU incorporation) was assessed in parallel experiments after 48 and 24 h, respectively (Figures 2A and 2B).

Treatment with crude, unfractionated MT-CM (which contains EVs as well as secreted proteins and other soluble factors) substantially enhanced myogenic differentiation (Figure 2B) as demonstrated by a >180% ($p < 0.001$) increase in MHC+ area (Figures 2B and 2C), >145% increase in myogenic index (Figure 2D), as well as a >230% ($p < 0.0001$) increase in the fusion index (Figure 2E), relative to the untreated control group. This pro-myogenic effect could not be explained by enhanced cell proliferation, as no significant difference in the total number of nuclei at DM2 (Figure 2F) or the proliferation index at DM1 (Figures 2B and 2G), was observed. Treatment with the EV-containing fraction (CM EVs) also enhanced myogenic differentiation (Figure 2B), resulting in an increase in the myogenic and fusion indices by >70% ($p < 0.01$) (Figure 2D) and ∼180% ($p < 0.0001$) (Figure 2E), respectively. A ∼75% increase in MHC+ area was observed, which is consistent with the other two measures of myogenic differentiation for this treatment fraction, although these effects did not reach statistical significance (Figure 2C). The pro-myogenic effect of the EV-containing CM fraction could not be explained by an increase in cell proliferation (Figures 2F and 2G).

In general, treatment with either of the EV-depleted fractions had minimal effect on myogenic differentiation. A relatively small (∼70%) increase ($p < 0.05$) in the fusion index was observed for the treatment group enriched in particles of 100–300 kDa (Figure 2E). This may be accounted for by indirect effects of increased cell density as a result of a greater number of nuclei observed only in this treatment group ($p < 0.05$) (Figure 2F). Furthermore, the proliferation index for this group was similarly increased, although this did not reach statistical significance at the $p < 0.05$ level (Figure 2G).

These findings suggest that differentiating MTs are capable of releasing pro-myogenic factors into the culture medium, which can be transferred to recipient cultures. Such differentiation-enhancing effects are observed for MT-derived unfractionated CM and in the EV-containing CM fraction, but not for EV-depleted fractions.

LC-purified EVs induce opposing effects on myogenic differentiation depending on dose

Given the pro-myogenic effects observed when MBs were treated with EV-containing MT-conditioned medium (CM), we next sought to determine if similar effects could be demonstrated for higher-purity EV preparations isolated by UF-size exclusion liquid chromatography (SEC).[28] To this end, EVs were purified from C2C12 myotubes (designated as MT-EVs) and undifferentiated C2C12 myoblasts (MB-EVs, which were *a priori* not expected to promote myogenic differentiation). The resulting highly pure EV preps were transferred to separate C2C12 MB cultures at the time of initiating differentiation (i.e., DM0) (Figure 3A). The protein content of each fraction was measured by UV absorbance at 280 nm to monitor the liquid chromatography process, and fractions collected accordingly (Figure 3B). EVs elute early as a relatively well-defined peak (fractions ∼5–10), and so these fractions were collected and pooled. EV isolates were characterized by nanoparticle tracking analysis (NTA), which showed that the modal sizes for MB-EVs and MT-EVs were 108.9 and 83.9 nm, respectively (Figure 3C), consistent with the expected size distributions for exosomes and other small microvesicles (i.e., ∼30–150 nm), and were not statistically different from one another (Student's t test, $p = 0.0793$). Western blot analysis of EV preparations demonstrated enrichment of the EV-specific markers, Alix (PDCD6IP), TSG101, and CD9, while the cell-specific marker, Calnexin (CANX), was only detected in lysates derived from the producer cells (Figure 3D). Analysis of EV preparations by transmission electron microscopy (TEM) revealed the presence of particles with characteristic EV-like morphology (Figure S2). These findings demonstrate the successful isolation of pure EVs derived from C2C12 MBs and MTs.

C2C12 cultures were treated according to Figure 3A with UF-SEC-purified EVs at doses ranging from 2×10^2 to 2×10^{11} particles/mL, and

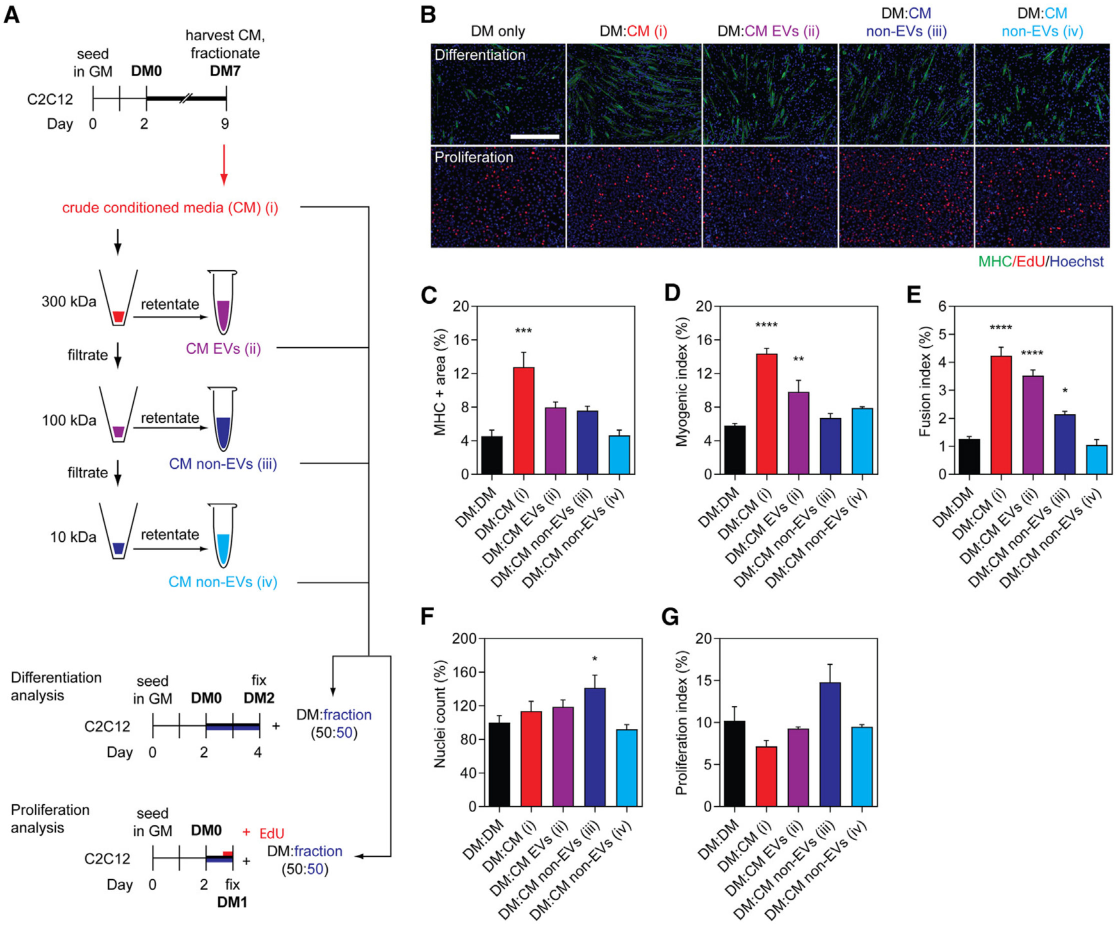

Figure 2. EV-containing myotube-conditioned medium enhances myoblast differentiation

(A) C2C12 cells were cultured in GM for 2 days and then switched to DM for 1 week. Myotube-conditioned medium (CM) from DM7 cultures was fractionated using sequential molecular weight (Mr) cutoff filters of 300, 100, and 10 kDa. Treatment fractions obtained included (1) crude CM, (2) EV-containing CM, and (3 and 4) EV-depleted CM. Recipient cultures were treated with a 50:50 mixture of fractionated CM and fresh DM. Treatment with an equal volume of DM was included as the DM-only control. Samples were collected after 48 h for analysis of myogenic differentiation, or after 24 h for proliferation analysis after pulsing with EdU. (B) Myogenic differentiation was assessed by MHC IF, and was quantified by (C) measuring the MHC+ area, and by calculating the (D) myogenic index and (E) fusion index. Proliferation was assessed by determining (F) the percentage of nuclei per representative field of view relative to the control group, and (G) the proliferation index based on EdU positivity. All microscopy images were taken at 10× magnification. Scale bar, 400 μm. All values are mean + SEM (n = 4). Statistical significance was determined by one-way ANOVA with Bonferroni post hoc test, *$p < 0.05$, **$p < 0.01$, ***$p < 0.001$, ****$p < 0.0001$.

myogenic differentiation assessed by MHC IF staining. The transfer of lower doses of MT-EVs (2×10^2 to 2×10^8 particles/mL) to MB cultures enhanced myogenic differentiation relative to the untreated controls (Figures 3E–3H). The greatest effect of the MB-EV treatment group was observed at the lowest dose (2×10^2 particles/mL), achieving a ∼30% increase ($p < 0.01$) in MHC+ area (Figure 3F), ∼35% increase ($p < 0.0001$) in myogenic index (Figure 3G), and ∼50% increase ($p < 0.001$) in the fusion index (Figure 3H). Very similar effects were observed when MBs were treated with MB-EVs, especially at doses 2×10^4 and less (Figures 3E–3H). Treatment with MT-EVs resulted in the most significant promotion of myogenic differentiation at 2×10^4 particles/mL dose, with a ∼25% increase in MHC+ area ($p < 0.01$) (Figure 3F), a ∼40% increase in myogenic index ($p < 0.001$) (Figure 3G), and a ∼55% increase in and fusion index ($p < 0.01$) (Figure 3H). Conversely, at the highest dose (2×10^{11} particles/mL) both MB-EVs and MT-EVs inhibited myogenic differentiation with a ∼25%–30% reduction in myogenic index ($p < 0.01$) (Figure 3G) and a ∼40% reduction in fusion index ($p < 0.01$) (Figure 3H). At the second highest dose tested (2×10^{10} particles/mL) there were no phenotypic effects for treatment with either MB-EVs or MT-EVs (Figures 3E–3I).

Nuclei numbers were unaffected by treatment with either MB-EVs or MT-EVs with one exception: treatment with MB-EVs at the highest dose resulted in a ∼25% ($p < 0.01$) increase in the total number of nuclei (Figure 3I). As such, the positive effects of

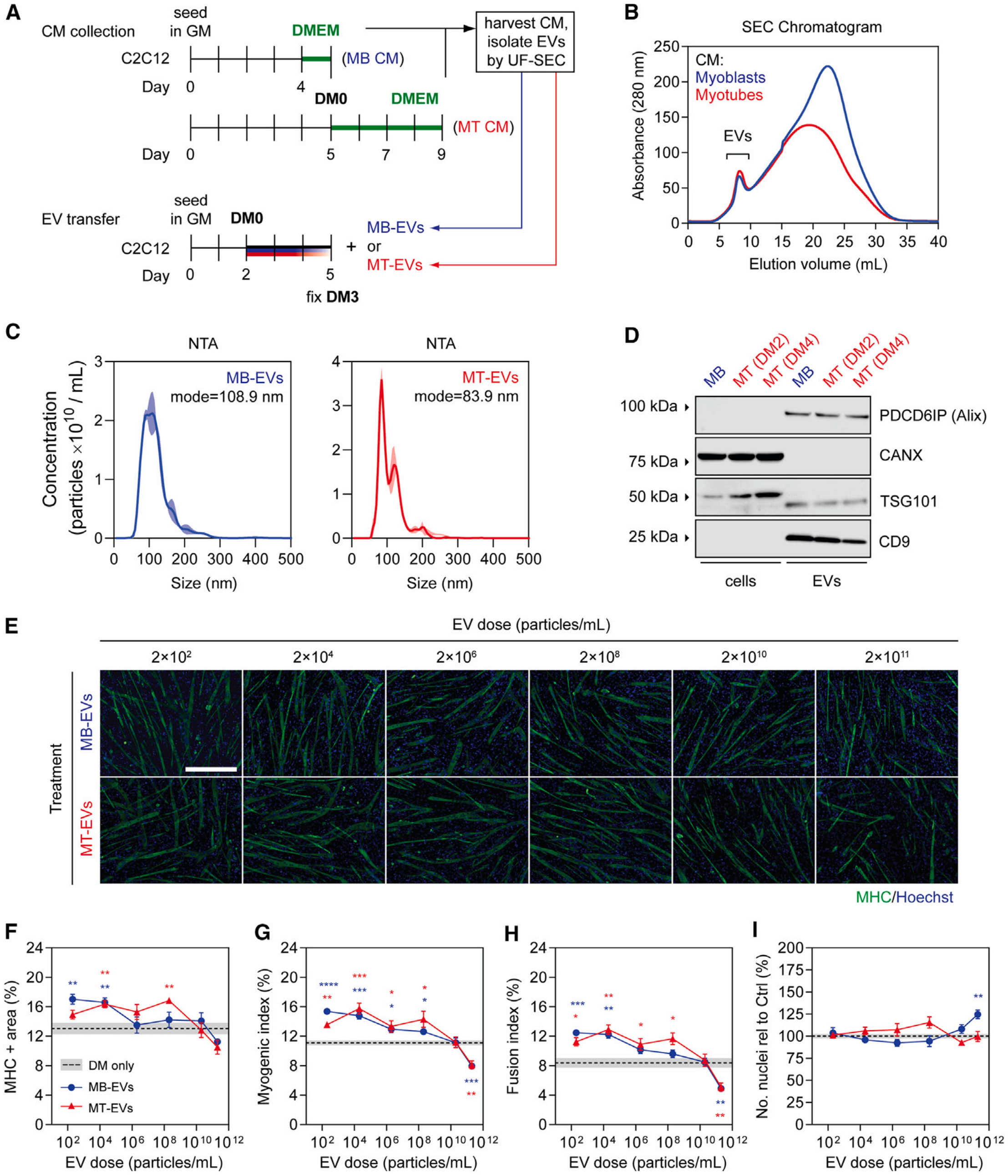

Figure 3. Effect of myoblast- and myotube-derived EVs on myogenic differentiation

(A) EVs were isolated by ultrafiltration-size exclusion liquid chromatography (UF-SEC) from CM obtained from C2C12 myoblasts (i.e., MB-EVs) or myotubes (i.e., MT-EVs). Myoblasts were grown in GM for 3 days and serum-free medium (DMEM) for 1 day. Myotubes were grown in GM for 5 days, and then differentiation induced by serum withdrawal. The medium was changed and EVs were collected in serum DMEM after a further 2 days. Recipient cultures were treated with MB-EVs or MT-EVs at doses as indicated. (B) The EV-containing eluates from the SEC column were identified by UV spectrophotometry absorbance at 280 nm, and pooled before treating C2C12 cells grown in GM for 2 days at the time of switching to DM. (C) The modal size and concentration of EVs was determined by nanoparticle tracking analysis (NTA). NTA curves represent the mean distribution from three separate isolations ± SEM (represented by the shaded area). (D) Exosome-specific (PDCD6IP/Alix, TSG101, and CD9) and cell-specific (CANX/Calnexin) markers were detected by western blot. (E) Myogenic differentiation was assessed by MHC IF at DM3 and quantified by (F) measuring the MHC+ area, and using (G) myogenic and (H) fusion indices. (I) The total number of nuclei per representative field of view are shown as a percentage relative to the control group. All microscopy images were taken at 10× magnification. Scale bar, 400 μm. Values are mean ± SEM (n = 4). Cultures treated with complete DM were used as the DM-only controls. Statistical significance was determined by a Student's t test relative to the control, *$p < 0.05$, **$p < 0.01$, ***$p < 0.001$, ****$p < 0.0001$. Significance indicators are colored corresponding to their respective comparisons (i.e., blue for MB-EVs vs. DM only, red for MT-EVs vs. DM only).

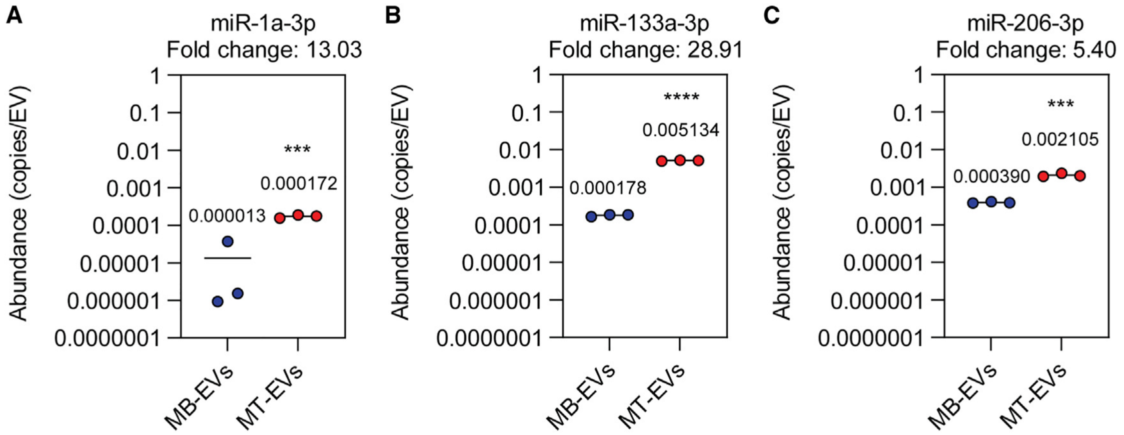

Figure 4. Determination of miRNA copies per vesicle in myoblast- and myotube-derived EVs

EVs were collected from myoblasts (MBs) or myotubes (MTs) as described in Figure 3A. Preparations were analyzed by NTA to determine particle counts, and absolute quantification small RNA TaqMan RT-qPCR to determine miRNA copy numbers was performed. The copies of miRNA per EV were calculated for (A) miR-1a-3p, (B) miR-133a-3p, and (C) miR-206-3p. Mean values are indicated (n = 3). Statistical significance was determined by a Student's t test, ***$p < 0.001$, ****$p < 0.0001$.

MB-EV and MT-EV transfer on myogenic differentiation at all of the lower doses therefore cannot be explained by alterations in cell density.

Interestingly, the degree of enhancement observed following EV treatment (Figures 3E–3H) was substantially lower than that observed for treatment with EV-containing CM in the previous experiment (Figures 2B–2E). Pro-myogenic effects in the former experiments are therefore likely the result of a combination of both EV-mediated and non-EV-mediated effects.

MyomiRs are scarce in both myoblast- and myotube-derived EVs

Our lab has previously reported that myomiRs are induced by differentiation, and present at very low levels in undifferentiated MB cultures.[6] It is therefore highly unlikely that the pro-myogenic effects observed with low-dose EV treatment were as a result of the functional transfer of EV-contained extracellular myomiRs, as such effects were observed with both MB- and MT-derived EVs.

To analyze the myomiR content of UF-SEC-purified EVs, we performed absolute quantification miRNA RT-qPCR on known quantities of MB-EVs and MT-EVs (determined by NTA). In this manner, the number of myomiR copies per EV could be determined. All three myomiRs (miR-1a-3p, miR-133a-3p, and miR-206-3p) were significantly upregulated ($p < 0.001$) in MT-EVs relative to MB-EVs (by 13-, 28.9-, and 5.4-fold, respectively) (Figure 4). For the MT-EVs, myomiRs were present at levels that were much lower than 1 copy per EV (miR-1a-3p, 1 copy per 5,814 EVs; miR-133a-3p, 1 copy per 195 EVs; and miR-206-3p, 1 copy per 475 EVs). These low abundance levels provide further evidence that the EV-associated pro-myogenic effect observed in C2C12 cultures is unlikely to be mediated by myomiRs.

Choice of collection medium influences pro-myogenic effects of transferred extracellular protein

We next sought to determine whether treatment with EV-depleted, soluble protein could induce pro-myogenic effects. A benefit of the UF-SEC technique is that non-EV protein-containing fractions can be collected in parallel with EV isolation using the same input CM. As such, the non-vesicular secreted protein fractions were collected from late stage (DM7) differentiating C2C12 MTs and transferred to MB cultures at the time of switching to DM (Figure 5A). Fifteen protein fractions were collected based on their temporal order of elution from the column, with protein separated such that the early fractions contain higher-Mr proteins and late fractions contain lower-Mr proteins (Figure 5B).

Recipient C2C12 cultures were treated with 1 μg/mL of collected material from a single representative fraction (no. 12). We observed that there was no effect on myogenic differentiation when C2C12 cultures were treated with fraction no. 12 protein collected in DMEM (as described above) (Figures 5C–5F). However, a profound increase in myogenic differentiation was observed when condition medium was collected in Opti-MEM (Figures 5C–5F). This was represented by statistically significant ($p < 0.0001$) increases in MHC+ area (>115%), myogenic index (>160%), and fusion index (>240%). The total number of nuclei were also increased in C2C12 cultures treated with protein fraction no. 12 collected in Opti-MEM by ∼15% ($p < 0.05$). Such pro-myogenic or pro-proliferative effects were not observed when cells were first treated with Opti-MEM and then DMEM subsequently used as an isolation medium. This suggests that the enhanced differentiation observed above is due to co-purification of a factor from the Opti-MEM itself, rather than the result of Opti-MEM promoting the secretion of a pro-myogenic signal. Similarly, no phenotypic effects were observed when donor cells were cultured in GM, and CM collected in DMEM (Figures 5C–5F).

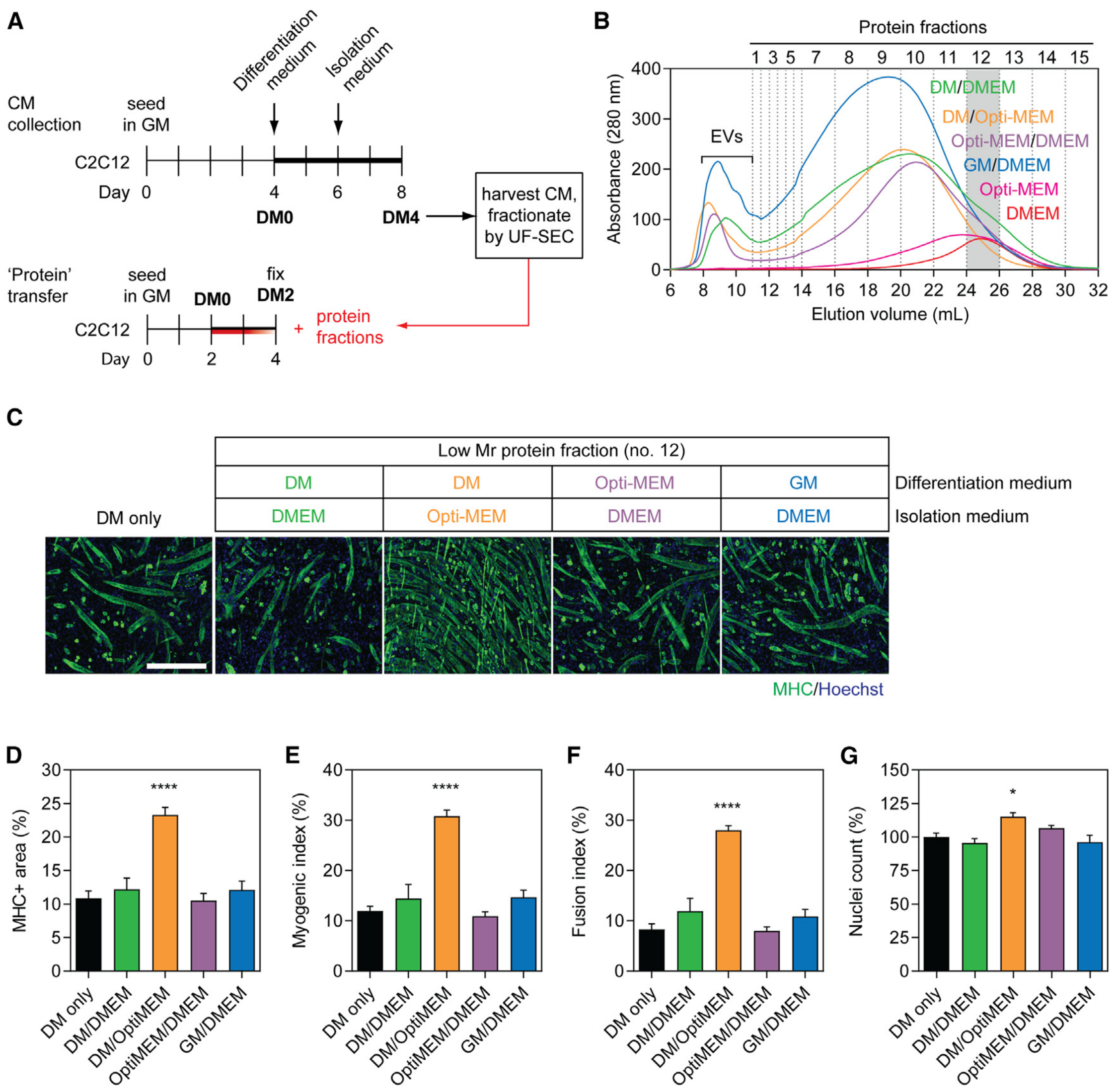

Figure 5. Pro-myogenic factors in Opti-MEM confound results from myotube-derived secreted protein transfer experiments

(A) C2C12 myotube-derived secreted protein fractions were isolated by UF-SEC from CM obtained from donor cultures with various combinations of differentiation medium and isolation medium: (1) DM followed by DMEM, (2) DM followed by Opti-MEM, (3) Opti-MEM followed by DMEM as indicated in the schematic, and (4) GM for 3 days followed by 1 day of DMEM. (B) The protein eluates from the SEC column were measured by UV spectrophotometry absorbance at 280 nm. Equal volumes of fresh Opti-MEM or DMEM were processed by SEC in parallel. C2C12 cells were grown in GM for 2 days and then treated with (C) 1 μg/mL of extracellular protein no. 12 from each set of culture conditions at the time of switching to DM. Untreated (DM only) cultures were included as negative controls. Myogenic differentiation was assessed by MHC IF at DM2 and quantified by (D) MHC+ area, (E) myogenic index, and (F) fusion index. (G) The total number of nuclei per representative field of view are shown as a percentage relative to the control group. All microscopy images were taken at 10× magnification. Scale bar, 400 μm. All values are mean + SEM (n = 4). Statistical significance was determined by one-way ANOVA with Bonferroni post hoc test, *$p < 0.05$, ****$p < 0.0001$.

We next investigated whether LC fractions from unconditioned DMEM and Opti-MEM medium could similarly promote myogenic differentiation. To this end, fresh DMEM and Opti-MEM were fractionated by UF-SEC and their 280 nm traces showed that they both exhibited broad peaks in the low-Mr range (fraction nos. 8–13) (Figure 5B). Differentiating C2C12 cultures were treated with isolated fraction no. 12 (1 μg/mL) collected from these fresh, unconditioned media. A strong pro-myogenic effect was observed with the protein fraction derived from Opti-MEM but not for DMEM (Figure S3). Treatment of C2C12 cultures with bovine serum albumin (BSA) (5 μg/mL) had no effect on myogenic differentiation or proliferation (Figure S4). Taken together, these data show that protein factions collected from Opti-MEM are sufficient to recapitulate the phenomenon observed with CM, and that this effect could not be explained by an increase in non-specific extracellular protein.

To investigate the Opti-MEM phenomenon further, C2C12 MBs were treated with each of the proteins fractions (collected in Opti-MEM, 1 μg/mL) at the time of switching to DM and effects on myogenic differentiation were assessed by MHC IF 24, 48, and 72 h post treatment (Figure S5A). All fractions were analyzed and fraction nos. 3 and 12 selected as representative of high- and low-Mr extracellular protein fractions, respectively (vertical gray bars on the SEC chromatogram in Figure S5B). The MHC+ area was progressively increased over time, and significantly increased ($p < 0.01$) at 48 and 72 h following treatment with the low-Mr protein fractions in the range of fraction nos. 7–15 (Figures S5C and S5D). This effect was greatest at 72 h post treatment, with fraction no. 12 resulting in a >145% increase in MHC expression relative to the control. The total number of nuclei was also significantly increased at each time point for treatment fraction nos. 7–15, peaking with no. 12 at >160% relative to the DM-only control (Figure S5E). Furthermore, cell proliferation was increased even at 24 h following treatment ($p < 0.05$) with fraction nos. 7–15, the maximal effect being a >160% increase in the number of nuclei. Conversely, an increase in myogenic differentiation was only apparent after 48 h post treatment. Comparatively smaller increases in myogenic differentiation (<45%) or proliferation (<30%) were observed in the high-Mr protein fractions (nos. 1–6) that elute immediately after the EV fractions relative to the DM-only control, with the majority of these not reaching statistical significance (Figures S5C–S5E, significance indicators not shown for convenience). These data indicate that one or more low-Mr extracellular proteins induces a profound increase in myogenic differentiation and cell proliferation when Opti-MEM is used as an isolation medium. Pro-myogenic effects were observed with multiple low-Mr protein fractions, suggesting that the active component protein(s) are broadly distributed across fractions. The maximal effect observed in fraction no. 12 corresponds with the distribution of Opti-MEM-derived proteins (Figure 5B). Notably, LC was performed using Sepharose 4 Fast Flow resin, which has a high separation range (4×10^4 to 3×10^7 Da) with respect to soluble proteins, and therefore it is expected that individual proteins will be broadly distributed across multiple fractions, which was consistent with experimental observation with respect to the 280 nm SEC trace observed for fresh, unconditioned Opti-MEM (Figure 5B) and for the phenotypic effects observed in Figures S5D and S5E.

The exact composition of Opti-MEM culture medium is proprietary; however, according to the product datasheet (Thermo Fisher Scientific) it contains 15 μg/mL of protein in total, which includes insulin and transferrin.[29] These factors are both incidentally also components of insulin transferrin selenium medium,[30] a supplement used in a number of studies to promote myogenic differentiation of cells *in vitro*.[31,32] To test possible confounding effects mediated by these cell culture medium additives, C2C12 cells were grown in GM for 2 days, and then treated with either insulin or transferrin (1 μg/mL) at the time of switching to DM (Figure 6A). The 1 μg/mL dose was selected to match the concentration of total extracellular protein transferred in the experiments described above (Figures 5 and S5). Myoblast cultures treated with insulin exhibited a pronounced enhancement in myogenic differentiation with increases in MHC+ area expression (>95%), myogenic index (>150%), and fusion index (>230%) at DM2 (Figures 6B–6E). Conversely, treatment with transferrin had no effect on myogenic differentiation. Assessment of the number of nuclei per well revealed that treatment with insulin resulted in a ∼150% increase ($p < 0.0001$) relative to the DM-only control (Figure 6F).

Vesicle-mediated pro-myogenic effects are influenced by EV isolation method

Given that we observed a confounding pro-myogenic effect of low-Mr extracellular protein fractions when using Opti-MEM as a collection medium (Figure 5), we next sought to investigate whether the choice of EV isolation method can also contribute toward this phenomenon. As such, CM was collected from late-stage differentiating MT cultures (DM4) in either Opti-MEM or DMEM, and then EVs isolated using either UF-SEC (designated as LC MT-EVs) or polymer precipitation (using the commercially available ExoQuick kit, designated as EQ MT-EVs) (Figure 7A). The protein content of each fraction obtained by UF-SEC was measured by UV absorbance at 280 nm (Figure 5B). EV samples from both isolation methods were characterized by NTA, indicating that the particle modal sizes were ∼71 and ∼97 nm for LC MT-EVs and EQ MT-EVs, respectively (Figure 7B), consistent with the expected sizes of exosomes and other small microvesicles. EQ MT-EVs were significantly larger than the corresponding LC EV treatment group (i.e., for both DMEM and Opti-MEM collection medium) (Student's t test, both $p < 0.05$).

LC MT-EVs and EQ MT-EVs were transferred to MB cultures at the time of initiating differentiation, and myogenic differentiation assessed after 2 days (Figure 7C). To test the potential confounding effects of Opti-MEM we selected a dose of 2×10^9 particles/mL, as this was expected to have no overall effect on myogenic differentiation as mediated by the EVs per se based on our previous findings (Figures 3E–3H). Treatment with EQ MT-EVs at this dose exhibited a highly pro-myogenic effect when isolated in the presence of Opti-MEM, with a >90% increase in both the MHC+ area and myogenic index relative to the DM-only control (Figures 7D and 7E), and a >280% increase in the fusion index (all $p < 0.0001$) (Figure 7F). Conversely, LC MT-EVs had no effect on myogenic differentiation, as expected. EVs collected in DMEM exhibited no significant effects on myogenic differentiation when purified by either method. The number of nuclei was not altered relative to the control for any of the treatment groups (Figure 7G).

This Opti-MEM-associated pro-myogenic effect was found to be dose dependent, with EQ MT-EV doses $\geq 2 \times 10^7$ particles/mL promoting enhanced differentiation (Figures S6A and S6B) in contrast to the results observed for LC MT-EVs (Figure 3). Taken together, these data strongly suggest that contaminating proteins (e.g., insulin) enhance myogenic differentiation in polymer-precipitated EV isolates when collected in Opti-MEM isolation medium.

DISCUSSION

Here, we have investigated the cell-to-cell signaling potential of EVs and non-vesicular extracellular protein in the context of myogenic

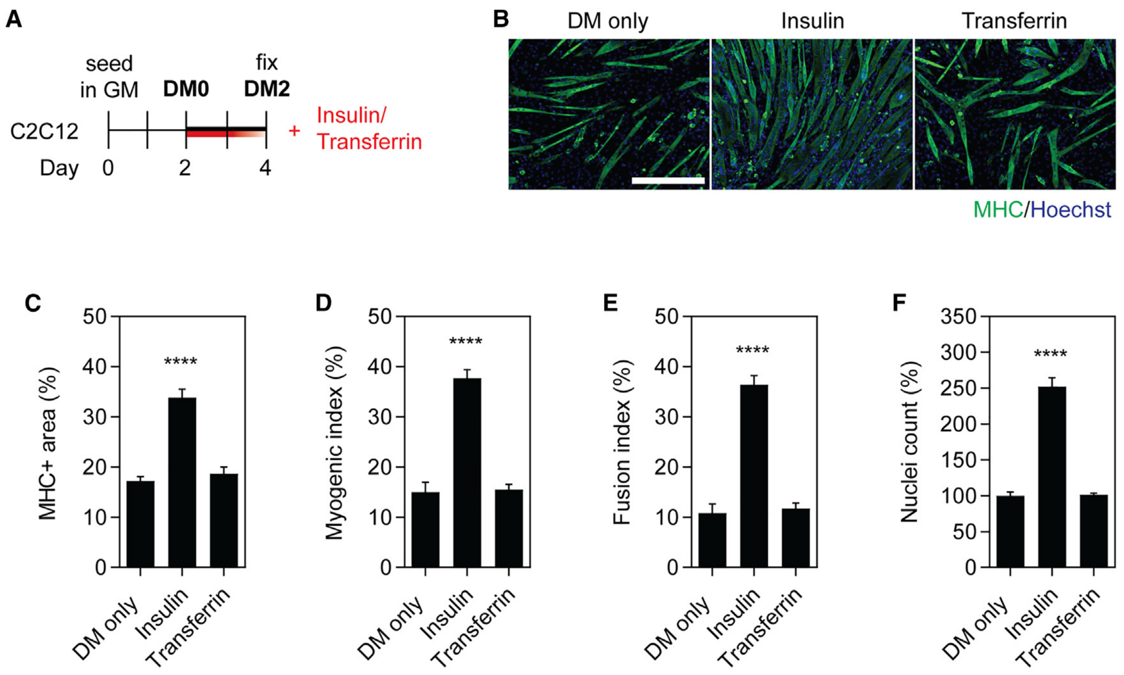

Figure 6. Insulin, but not transferrin, enhances myogenic differentiation in C2C12 cell cultures

(A) C2C12 myoblasts were grown in GM for 2 days, and treated with insulin or transferrin (1μg/mL) at the time of switching to DM for 2 days. Myogenic differentiation was assessed by MHC IF at DM2 (B), and quantified by measuring the (C) MHC+ area, (D) myogenic index, and (E) fusion index. Untreated (DM only) cultures were included as negative controls. (F) The total number of nuclei per representative field of view are shown as a percentage relative to the control group. All microscopy images were taken at 10× magnification. Scale bar, 400 μm. Values are mean + SEM (n = 4). Statistical significance was determined by a one-way ANOVA with Bonferroni post hoc test, ****$p < 0.0001$.

differentiation. This study adds to the growing literature that supports the notion that EVs can mediate a pro-myogenic effect, at least to some extent. We observed that inhibition of EV release using GW4869 (a small molecule inhibitor of neutral sphingomyelinase 2 [nSMase2] that inhibits the ceramide-dependent exosome biogenesis pathway)[33] resulted in suppression of myogenic differentiation (Figure 1). In addition, siRNA-mediated knockdown of *Rab27a* and *Rab27b*, two RAB GTPases that are involved in the ESCRT-dependent mode of exosome release,[27,34–36] recapitulates this effect (Figure S1). Similarly, inhibition of EV uptake via treatment with heparin also inhibited myogenic differentiation (Figure 1). Conversely, myotube-CM, and CM that had been filtered to retain EVs, promoted myogenic differentiation in recipient C2C12 cultures (Figure 2). Such CM preparations contain a complex mixture of biomolecules and so pro-myogenic effects could not be unambiguously attributed to EVs for this experiment. To further investigate the role of EVs specifically, we utilized UF-SEC to obtain highly pure EV preparations (Figure 3). Treatment of myoblast cultures with UF-SEC-purified EVs enhanced myogenic differentiation, but only at concentrations of 2×10^8 particles/mL and below. Conversely, differentiation was inhibited at the high dose (2×10^{11} particles/mL, corresponding to ∼1 mg/mL of protein). These surprising findings highlight the importance of determining appropriate dose windows, as opposite phenotypic outcomes were observed in this biological context depending on the dose used. Notably, recently it has been reported that treatment with high concentrations of exogenous EVs can inhibit endogenous EV production.[37]

Determining physiologically relevant EV doses remains a significant challenge. Forterre et al. conducted proteomic analysis of EVs produced by C2C12 myoblasts and myotubes, and reported that differentiating C2C12 cells release 0.42 ± 0.01 μg/mL of EVs per 24 h period.[15] This value was therefore considered to be an approximate "physiological dose" for C2C12 cells in this study. Many of the studies that have assessed the functional transfer of EVs in culture used protein concentration as a measure of dose, and phenotypic effects have been reported with 10–200 μg/mL of exogenously derived EVs.[1,19,38] Importantly, many of the publications describing the functional role of EVs in myogenic differentiation[13,15,18–20,39,40] have utilized commercially available polymer precipitation kits,[19] that are known to co-purify non-vesicular proteins and soluble factors.[41] Ultracentrifugation is generally regarded as the gold standard method of EV isolation and has also been used to purify myocyte-derived EVs,[1,38] but presents its own technical issues. Specifically, ultracentrifugation results in inconsistent yields and low purity as a result of co-sedimentation of non-vesicular soluble proteins and RNAs with EVs.[28,42,43] As a result, the use of protein concentration as a measure of dose may be misleading when comparing between studies that utilize distinct EV isolation methods. With this limitation in mind, this study used MB-EVs and MT-EVs ranging from 2×10^2 to 2×10^{11} particles/mL

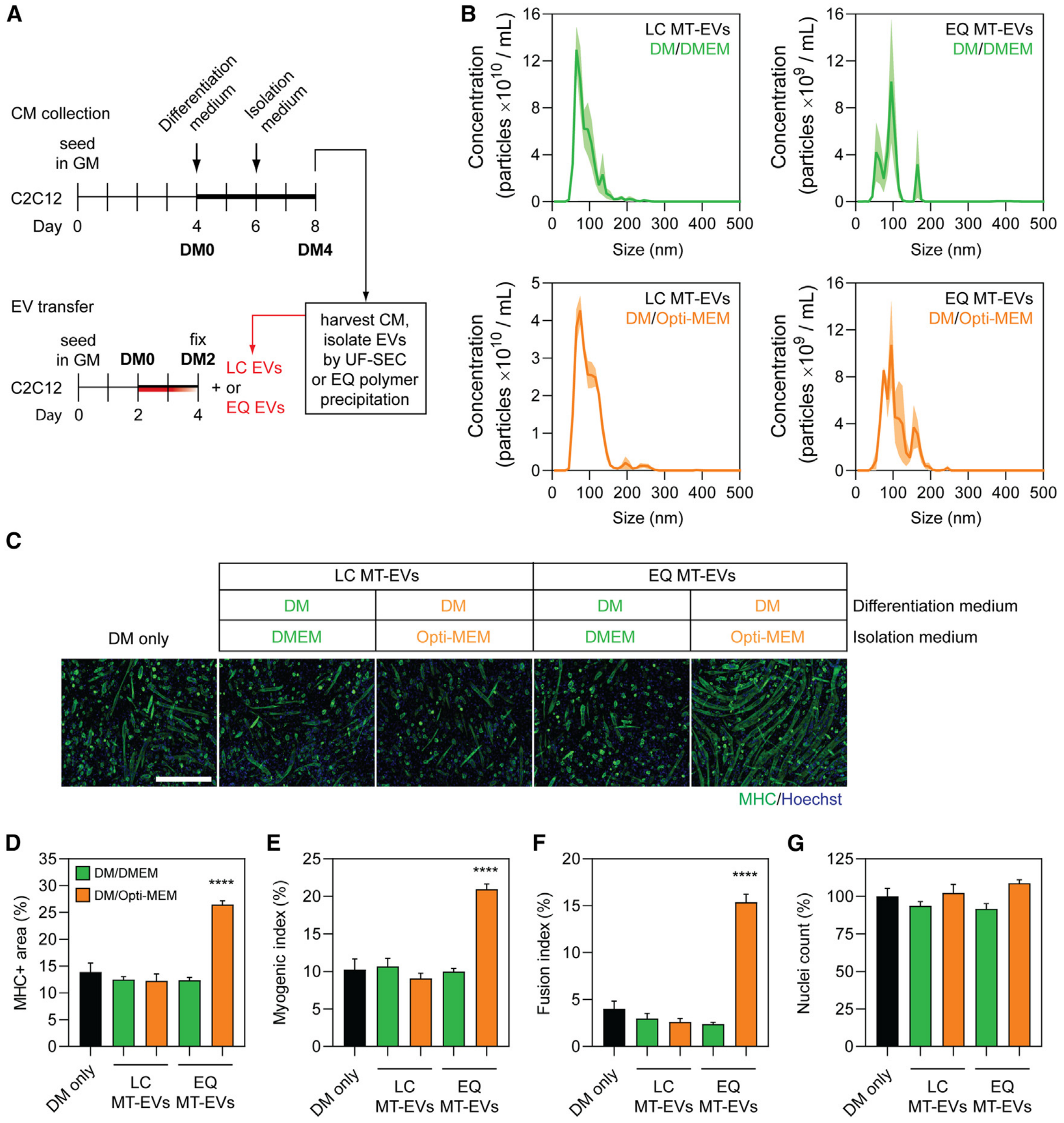

Figure 7. EVs obtained from Opti-MEM collection medium exhibit substantial pro-myogenic effects only when using polymer precipitation isolation methods

(A) EVs were isolated by UF-SEC (LC MT-EVs) or ExoQuick polymer precipitation (EQ MT-EVs) from C2C12 myotube-derived CM obtained from cell growth in different isolation medium. Myoblasts were grown in GM for 4 days, DM for 2 days, and isolation medium (Opti-MEM or DMEM) for a further 2 days. Recipient C2C12 myoblast cultures were treated with 2×10^9 EV/mL at the time of switching to DM. (B) The modal size and concentration of EVs was determined by NTA. Values are the mean distribution from three separate experiments, shown ± SEM (the shaded area). (C) Myogenic differentiation was assessed in recipient cultures by MHC IF at DM2 and quantified by (D) measuring the MHC+ area, and using (E) myogenic and (F) fusion indices. (G) The total number of nuclei per representative field of view are shown as a percentage relative to the control group. Untreated (DM only) cultures were included as negative controls. All microscopy images were taken at 10× magnification. Scale bar, 400 μm. All values are mean + SEM (n = 4). Statistical significance was determined by a one-way ANOVA with Bonferroni post hoc test, ****$p < 0.0001$.

(corresponding to 1 pg/mL to 1 mg/mL of protein). Notably, phenotypic effects were observed with very low levels of EVs (2×10^2), which argues against the transfer of EV contents as an underlying mechanism. EV surface signaling effects may provide an alternative explanation. Conversely, a small amount of EV transfer (or surface interaction) may trigger release of a paracrine factor by the recipient cell, thereby modulating the behavior of the whole culture.

Our group,[6,14] and others,[18,20] have hypothesized that the protection of ex-myomiRs within EVs may enable their cell-to-cell transfer in a

paracrine manner. However, in this study we observed pro-myogenic effects using EVs harvested from both differentiated and undifferentiated donor cultures. Given that myomiR levels are lowly expressed in MB donor cultures,[6] this finding strongly argues against a myomiR-related mechanism for the observed pro-myogenic effects. Furthermore, EV myomiRs were similarly found to be present at very low levels (<1 copy per 195 EVs for miR-133a-3p in MT-EVs) (Figure 4), consistent with reports of EV miRNA concentrations in other biological contexts.[44,45] Similarly, we have previously reported that the majority (~99%) of extracellular myomiRs are non-vesicular in serum derived from the dystrophin-deficient *mdx* mouse,[6,9,14] and are instead stabilized in soluble protein complexes. We were therefore motivated to determine if transfer of the non-vesicular majority of myomiRs could induce changes in C2C12 phenotypes. To this end, we collected non-vesicular protein fractions by UF-SEC in parallel with EV isolation. When the protein fractions were collected in serum-free DMEM medium, no pro-myogenic effects were observed (Figure 5). These data suggest that extracellular protein-associated myomiRs cannot promote myogenic differentiation in this model system. However, when Opti-MEM was utilized as the isolation medium, the resulting protein fractions greatly enhanced myogenic differentiation (Figures 5 and S5). Protein purified from fresh Opti-MEM and treatment with insulin (a major component of Opti-MEM) were sufficient to recapitulate this effect (Figures 6 and S3). Together these data strongly suggest that the use of Opti-MEM as an isolation medium is a major potential confounding factor in studies of CM transfer in the context of myogenic differentiation (or any other insulin-sensitive biological system).

The use of polymer precipitation methods makes it difficult to conclusively attribute a functional effect observed to EV-specific paracrine signaling rather than some other extracellular contaminant. Indeed, an International Society for Extracellular Vesicles position paper has specifically highlighted the problematic nature of polymer precipitation methods in EV research as these kits "do not exclusively isolate EVs, and are likely to co-isolate other molecules, including miRNA-protein complexes."[46] For instance, polymer precipitation methods of EV purification have been shown to co-purify vesicle-free miRNAs from rat plasma[47] and non-vesicular extracellular Argonaute-2 complexes from the secretome of MCF-7 breast cancer cell cultures.[43] Furthermore, it has been suggested that the use of EV precipitation techniques has led to the misattribution of pro-angiogenic and regenerative cell signaling effects as a result of the co-purification of non-vesicular soluble factors expressed by MSCs.[48] The data presented in this study provide evidence to support this notion, as polymer precipitated-EVs exhibited a pro-myogenic effect only when Opti-MEM was used as the collection medium (Figure 7). In contrast, LC-purified EVs collected in Opti-MEM did not induce similar pro-myogenic effects. These findings suggest that polymer precipitation methods co-purify medium contaminants with the potential to elicit profound confounding effects. However, it has recently been reported that a protein corona forms around EVs in the circulation.[49] It is therefore possible that techniques such as UF-SEC may strip away corona proteins that are important for the phenotypic effects mediated by EVs. However, it is doubtful that exogenous insulin at supraphysiological concentrations would be considered a bona fide EV corona protein, and so such an eventually is unlikely to explain the results presented here.

The endocrine system plays an important role in skeletal muscle metabolism through the targeted action of receptor-mediated signaling by growth factors (e.g., insulin-like growth factors 1 and 2), and hormones (e.g., growth hormone, insulin, and androgens).[50] Insulin is a low-Mr protein (5.8 kDa) that plays an important role in skeletal muscle function[51] via intracellular signaling cascades involving the PI3K/Akt and Shc/Ras/MAP kinase axes.[52] Such pathway activation has also been reported following insulin treatment in C2C12 myoblast cultures.[53,54] Notably, the dose of insulin used in the present study (i.e., at micromolar concentrations) is much higher than the range of circulating insulin in the body (i.e., at picomolar concentrations).[55]

While polymer precipitation methods may confound the biological interpretation of EV transfer experiments through the co-precipitation of medium-derived contaminants like insulin, they may equally concentrate endogenously derived factors. For example, a study by Hu et al. reported that IL-6 (a soluble cytokine that signals via interaction with its cognate receptor at the membrane of recipient cells) is packaged within EVs that are then capable of cell-to-cell communication in C2C12 myotubes and 3T3-L1 adipocytes based on polymer precipitation experiments.[56] The authors' own data do not support this conclusion, given that the signaling effect was blocked by anti-IL-6 antibodies. Instead, it is much more likely that soluble IL-6 was co-purified together with the vesicles, and the reported biological effects have been misattributed to the EVs themselves. Similarly, a study by Hettinger et al. used polymer precipitation to purify EVs from primary human myoblasts after the induction of senescence via treatment with hydrogen peroxide to suggest that EVs are senescence-associated secretory phenotype (SASP) mediators.[57] The SASP is a well-described effect associated with the release of multiple soluble extracellular factors (e.g., cytokines, proteases, growth factors, etc.) from senescent cells,[58] proteins which are likely to be co-purified in polymer-precipitated EV isolates.

The data presented here add to the growing body of literature suggesting that EVs constitute a secretory signaling function in skeletal muscle, although with several caveats. Forterre et al. reported that MT-EVs are capable of transferring exogenous small RNAs (siRNA and non-mammalian miRNA) to myoblasts, and suggested that EV-mediated transfer of miRNA-133a-3p can suppress *Sirt1* expression in recipient cultures.[40] In a follow-up study, the same group reported that MT-EVs (but not MB-EVs) suppressed myoblast proliferation.[15] This phenotypic finding was not observed in our data, although our experiments were not primarily designed to test this directly. Both of these studies utilized ultracentrifugation for EV isolation.

Several studies have reported pro-myogenic effects of EVs *in vivo*. Nakamura et al. reported that MSC-derived EVs (isolated by

ultracentrifugation) enhanced C2C12 differentiation and improved regeneration after cardiotoxin-induced muscle injury.[20] Similarly, Choi et al. reported that EVs (polymer precipitated from medium containing 10 μg/mL insulin) derived from differentiating human skeletal myoblasts could promote myogenic differentiation in human adipose-derived stem cells and reduced fibrosis in a laceration muscle injury model.[19] Multiple other studies have utilized polymer precipitation in the context of EV-mediated cell-to-cell signaling and myo-miR transfer *in vivo*.[18,39]

In conclusion, this study demonstrates that highly pure preparations of EVs derived from differentiating muscle cells have the potential to transfer pro-myogenic signals to myoblasts when provided at appropriate concentrations. Notably, opposite phenotypic outcomes were observed at the highest dose of EVs administered, underlining the importance of dose-response experiments in EV research. Conversely, we observed that non-vesicular protein fractions were unable to transfer pro-myogenic signals to recipient cells provided that specific protein contaminants are absent. These findings are important as there is a risk that biological effects could be misattributed to EVs when they are in reality the result of exogenous or endogenous non-vesicular factors that are co-purified when using certain methods of EV isolation.

We propose that the use of Opti-MEM for sample collection should be avoided (or at least carefully controlled) for studies of EVs and CM transfer in myogenic cultures (and potentially in other insulin-sensitive biological systems). Similarly, polymer precipitation methods of EV isolation should be avoided due to the risk of introducing such confounding factors.

MATERIALS AND METHODS

Cell culture

C2C12 *Mus musculus* myoblast cells (obtained from ATCC, cat. no. CRL-1772) were maintained in growth medium (GM) (DMEM supplemented with 15% FBS and 1% antibiotic-antimycotic, all Thermo Fisher Scientific, Abingdon, UK). Myogenic differentiation was initiated by switching to low serum DM (DMEM supplemented with 2% horse serum and 1% antibiotic-antimycotic, all Thermo Fisher Scientific).

For typical experiments, C2C12 myoblasts were seeded at a density of 1×10^5 cells in 0.5 mL of GM per well in a 24-well multiplate, and cultured for 2 days. Subsequently, cultures were switched to DM and treated as appropriate.

EV isolation was performed on cultures grown in vesicle-free isolation medium using either DMEM supplemented with 1% antibiotic-antimycotic (hereafter DMEM), or Opti-MEM I Reduced Serum Medium (Thermo Fisher Scientific, cat. no. 31985062) supplemented with 1% antibiotic-antimycotic (hereafter Opti-MEM) as appropriate.

Small-molecule and protein treatments

For small-molecule treatments, cells were treated at the time of switching to DM with 10 μM of GW4869 (Sigma-Aldrich, Gillingham, UK), an inhibitor of nSMase2 in DMSO (Sigma-Aldrich), to impair EV biogenesis,[25] or 10 μM of heparin sulfate in water (Sigma-Aldrich) to prevent EV uptake or re-uptake.[26] For treatment with proteins, cells were treated at the time of switching to DM with bovine serum albumin, BSA (Thermo Fisher Scientific), insulin (from bovine pancreas, Sigma-Aldrich), or human transferrin (Sigma-Aldrich). BSA was used at a final concentration of 5 μg/mL, and insulin and transferrin were used at final concentrations of 1 μg/mL. Untreated cultures (DM only) and/or cultures treated with DMSO were included as controls.

RNA interference

Cells were treated with 100 nM of ON-TARGETplus Mouse *Rab27a* (11891) or *Rab27b* (80718) siRNA SMARTpools (Dharmacon, Lafayette, CO) complexed with Lipofectamine RNAiMAX (Thermo Fisher Scientific) according to the manufacturer's protocols. Transfection complexes were prepared in DMEM containing no supplements (Thermo Fisher Scientific). Target regions of the ON-TARGETplus SMARTpool siRNAs are listed in Table S1. ON-TARGETplus Non-targeting Pool (Dharmacon) was used as a negative siRNA control.

IF

IF was performed as described previously.[59] In brief, cell cultures were washed with phosphate-buffered saline (PBS) (Thermo Fisher Scientific), fixed with 4% paraformaldehyde (Santa Cruz Biotechnology, Dallas, TX), permeabilized with 0.25% Triton X-100 (AppliChem, Darmstadt, Germany), and blocked with 5% BSA (Sigma-Aldrich, St. Louis, MO). Cells were subsequently incubated sequentially with primary and secondary antibodies as appropriate (Table S2). Nuclei were stained with Hoechst stain (i.e., Hoechst 33258 Pentahydrate (bis-Benzimide)) (Sigma-Aldrich).

Microscopy images were acquired using an EVOS FL Cell Imaging Fluorescence Microscope (Thermo Fisher Scientific), with a 10×/0.25 objective lens. Images were captured using the inbuilt camera and subsequent image handling was performed in ImageJ.

Myogenic differentiation was quantified using three parameters. (1) Calculation of MHC+ area as a percentage of the total image area using a custom ImageJ script. This metric is convenient for the rapid processing of a large number of microscopy images as an initial screen. (2) The myogenic index was defined as the percentage of nuclei contained within all MHC+ cells. (3) The fusion index was defined as the percentage of nuclei contained within myotubes that contain three or more nuclei. Analyses were performed on four representative fields of view.

EdU incorporation assay

Cell proliferation was assessed 24 h post treatment using the Click-iT EdU Alexa Fluor 555 Imaging Kit (Thermo Fisher Scientific) according to the manufacturer's instructions. The cultures were pulse incubated with 10 μM EdU (5-ethynyl-2′-deoxyuridine) for 1 h to metabolically label newly synthesized genomic DNA in cells undergoing S-phase. Cells were subsequently fixed and permeabilized, followed by conjugation of Alexa Fluor 555-azide to the alkyne groups of the

EdU nucleotides by click chemistry. The proliferation index was calculated as the percentage of Alexa Fluor 555-positive nuclei.

RT-qPCR

Reverse transcriptase quantitative polymerase chain reaction (RT-qPCR) was conducted following the MIQE (minimum information for publication of quantitative real-time PCR experiments) guidelines[60] where possible or appropriate. RNA extraction from cells was performed using the Maxwell RSC simplyRNA Tissue Kit (Promega, Madison, WI) according to the manufacturer's instructions. RNA extraction from EVs was performed using TRIzol LS Reagent (Thermo Fisher Scientific) according to the manufacturer's instructions.

RNA concentrations were quantified by absorbance at 260 nm using a NanoDrop 2000 spectrophotometer (Thermo Fisher Scientific), and cDNA generated using the High-Capacity cDNA Reverse Transcription Kit (Thermo Fisher Scientific). cDNA templates were amplified on a StepOne Plus real-time PCR Thermocycler (Applied Biosystems, Waltham, MA) using Power SYBR Green Master Mix (Thermo Fisher Scientific) and gene-specific primers (Table S3). Duplicate technical replicates were performed per biological sample. Each reaction consisted of 2 μL of undiluted cDNA, 10 μL of 2× SYBR Green Master Mix, 1 μL of a 20× primer mix, and nuclease-free water to a final volume of 20 μL. Cycling conditions were as follows: initial denaturation for 10 min at 95°C, followed by 40 cycles of 15 s at 95°C and 1 min at 60°C. Melt curve analysis was performed after completion of the cycling protocol (temperature range 60°C–95°C, at a ramp rate of 0.3°C per second). A single peak confirmed the absence of primer-dimer products. A no template control (where water was substituted for the cDNA) was included for each assay run. Transcript levels were analyzed using the Pfaffl method.[61] *Rab27a* and *Rab27b* transcript levels were detected using gene-specific primers and normalized to the stable murine reference gene *Rplp0*, and data were scaled such that the mean of the control group was returned to a value of 1.

Absolute quantification miRNA RT-qPCR

miRNA RT-qPCR was performed as described in detail previously.[62–64] In brief, RNA was extracted from 1×10^{10} EV using TRIzol LS (Thermo Fisher Scientific) according to manufacturer's instructions with minor modification as described previously.[62] Three microliters of 5 nM synthetic miRNA mimic (cel-miR-39) was added to each sample at the phenolic extraction step as an exogenous spike control. Total RNA (5 μL) was then reverse transcribed using a pool of miRNA-specific stem loop primers and the TaqMan MicroRNA Reverse Transcription Kit (Thermo Fisher Scientific) according to manufacturer's instructions. Resulting cDNA (2 μL) was used per subsequent qPCR reaction (20 μL total volume). qPCR amplification was performed on a StepOne Plus real-time PCR Thermocycler with TaqMan Gene Expression Master Mix (Thermo Fisher Scientific) using universal cycling conditions: 95°C for 10 min, followed by 45 cycles of 95°C for 15 s, 60°C for 1 min. Details for all small RNA TaqMan assays are listed in Table S4. Notably, we have previously shown that the Small RNA TaqMan assay for miR-133a-3p is incapable of distinguishing between miR-133a-3p and miR-133b-3p.[65] Results are presented for miR-133a-3p with this caveat. Absolute quantification was performed by comparing samples with standard curves consisting of serial dilutions of synthetic miRNA mimics for the miRNAs of interest (Table S5). Copy numbers determined in this manner were further scaled according to the levels of cel-miR-39 to account for differences in extraction efficiency between samples.

TEM

Quality and purity of EV-containing samples (isolated by different methods) were evaluated by TEM. In brief, 10 μL of EV sample was applied to freshly glow discharged carbon Formvar 300 mesh copper grids (Agar Scientific, London, UK) for 2 min. The grid was then blotted dry with filter paper and stained with 2% uranyl acetate for 10 s. The water droplet was then removed and the grid was air dried for 15 min. Grids were imaged using a FEI Tecnai 12 TEM at 120 kV using a Gatan OneView CMOS camera (Gatan, Pleasanton, CA).

Western blot

EVs and EV producer C2C12 cells were lysed in RIPA buffer (both Thermo Fisher Scientific) and protein concentrations were determined using the Micro BCA Protein Assay Kit (Thermo Fisher Scientific) according to the manufacturer's instructions.

Protein was added to 1× NuPAGE LDS Sample Buffer and 1× NuPAGE Reducing Agent (both Thermo Fisher Scientific) in water and incubated at 75°C for 10 min for protein denaturation. Protein samples (10 μg) were loaded per well, and separated by SDS-PAGE using NuPAGE 4% to 12% Bis-Tris gel, 1.0 mm, Mini Protein Gels and the gel run at 100 V for 75 min in NuPAGE MOPS SDS Running Buffer (all Thermo Fisher Scientific). The proteins were transferred onto an Immobilon-fl polyvinylidene difluoride membrane (Sigma-Aldrich) at 100 V for 1 h on ice in NuPAGE Transfer Buffer (Thermo Fisher Scientific).

Membranes were blocked for 1 h at room temperature in Intercept (PBS) Blocking Buffer (LI-COR Biosciences, NE). Primary and secondary antibody incubations were carried out in Intercept Blocking Buffer supplemented with 0.1% Tween 20 (Sigma-Aldrich) overnight at 4°C, or for 1 h at room temperature, respectively. Blots were washed with PBS supplemented with 0.1% Tween 20 after antibody incubations. Details of antibodies are described in Table S2.

Ultrafiltration of myotube-CM

C2C12 cells were seeded at a density of 4.7×10^5 cells in 10 cm plates in 10 mL of GM and switched to DM 48 h later. The DM was changed 72 h later and the CM collected after a further 72 h from highly differentiated myotubes. The CM was centrifuged at 300 × *g* for 5 min at 4°C to remove extracellular debris. The supernatant was transferred to a fresh 50 mL tube and centrifuged at 2,000 × *g* for 10 min at 4°C to pellet the remaining larger, non-vesicular particulate matter. This was then passed through a 0.22 μm filter. The resulting CM sample was then further fractionated by

sequential UF using Mr cutoff filters as follows: 6 mL of the crude CM was passed through a 300 kDa Vivaspin concentrator (Sartorius AG, Göttingen, Germany) by centrifugation at 3,000 × *g* for ∼15 min (or until the retentate was ∼300 μL). The EV-containing fraction (CM-EVs) was obtained from the retentate and the volume of the flowthrough (FT) was increased to 3 mL in DM before filtration through a 100 kDa Amicon Ultra-15 centrifugal unit (MilliporeSigma, MA) by centrifugation at 3,000 × *g* for ∼10 min. The EV-depleted fraction containing particles and soluble factors of 100–300 kDa in size was obtained from the retentate and the volume of the FT was increased to 3 mL in DM once again before filtration through a 10 kDa Amicon Ultra-15 centrifugal unit (MilliporeSigma) by centrifugation at 3,000 × *g* for ∼10 min. The EV-depleted fraction containing particles and soluble factors of 10–100 kDa in size (e.g., growth factors and cytokines) was obtained from the retentate. Filters were not washed between fractionation steps to minimize the loss of material. In summary, treatment fractions obtained by spin filtration included (1) crude unfractionated CM, (2) CM containing EVs and other particles >300 kDa in size, and CM depleted of EVs with particles that are (3) 100–300 kDa or (4) 10–100 kDa in size. Treatment of recipient C2C12 myoblasts was performed immediately following isolation of CM fractions.

Isolation of EVs by UF-SEC

C2C12 myoblasts were seeded at a density of 4.7×10^5 cells in ten 15 cm plates (Thermo Fisher Scientific), each containing 20 mL of GM. For the collection of MB-CM, cells were cultured in GM for 4 days, and then switched to serum-free medium (DMEM containing antibiotics/antimycotics, hereafter referred to as DMEM) for a further 24 h. For the collection of MT-CM, cells were differentiated for 2 days, followed by a further 2 days in serum-free isolation medium (DMEM only).

In the cases of both MB and MT donor cultures, ∼200 mL of pooled CM was transferred into eight 50 mL tubes (Thermo Fisher Scientific) and centrifuged at 300 × *g* for 5 min at 4°C. The supernatant was then transferred to fresh tubes and centrifuged again at 2,000 × *g* for 10 min at 4°C. The supernatant was then pooled and filtered using a 0.22 μm filter unit. The resulting CM was kept at 4°C until ready for EV isolation.

UF-SEC was performed as described previously.[28] In brief, CM UF and concentration was carried out by tangential flow filtration using a Vivaflow 50 R 10,000 MWCO Hydrosart membrane (10 kDa Mr cutoff) (Sartorius, Göttingen, Germany) and a Masterflex L/S Easy Load machine (Antylia Scientific, St. Neots, UK). Typically, ∼200 mL of CM was concentrated to ∼15 mL by TFF. The volume was then further concentrated to <2 mL using a 10 kDa Amicon Ultra-15 centrifugal unit (Sigma-Aldrich).

SEC was carried out using the ÄKTA Prime instrument (GE Healthcare, Pollards Wood, UK) equipped with an ultraviolet flow cell. The concentrated CM was loaded onto a column packed with Sepharose 4 Fast Flow resin (GE Healthcare, IL). Eluates were measured at 280 nm absorbance to monitor the protein content of each fraction. The volume of each EV-containing fraction was set at 0.5 mL, and fractions 5–10 (EV containing) were pooled prior to downstream analysis to obtain MB- and MT-EVs. The total volume was concentrated to ∼2 mL by spin filtration using an Amicon Ultra-2 Centrifugal Filter Unit with Ultracel-100 membrane (100 kDa Mr cutoff) (Sigma-Aldrich). The non-vesicular protein fraction volumes were each collected in 2 mL of PBS, and were used without further processing in downstream experiments.

Polymer precipitation of EVs

Conditioned medium was collected from C2C12 cultures as described above and centrifuged at 300 × *g* for 5 min at 4°C to pellet, supernatant was collected and then centrifuged at 2,000 × *g* for 10 min at 4°C to remove any cellular debris, and then filter sterilized (0.22 μm). The processed CM (200 mL) was concentrated by TFF (10 kDa membrane) to a final volume of ∼15 mL for each sample. To precipitate EVs, 1 mL of ExoQuick-TC exosome precipitation solution (Stratech, Ely, UK) was added to 5 mL of concentrated CM, vortexed briefly, and then samples precipitated overnight at 4°C. Samples were then centrifuged at 1,500 × *g* for 30 min at 4°C to pellet EVs. The supernatant was discarded and the pellet resuspended in sterile PBS.

NTA

NTA was performed to determine the concentration and size range of EVs obtained by UF-SEC using a NanoSight NS500 analyzer using NTA 2.3 software (both Malvern Instruments, Worcestershire, UK). Measurements were analyzed using the following settings: the camera level was set at 14 and the detection threshold set at 5. All other post-acquisition settings were automated. Samples were diluted 1:2,000 for count detection within the range of 2×10^8 to 2×10^9 particles/mL. Particles were counted and the mean of triplicate 30 s recordings was obtained using an automated script.

CM and EV transfer treatments

For CM transfer experiments, recipient cells were treated at the time of switching to DM with a 50:50 mixture of 0.5 mL CM treatment fraction and 0.5 mL of fresh DM. Fresh medium was added so that recipient cultures were not deprived of nutrients that may have been depleted by the metabolic activity of the producer cells, or by the sequential filtration of the CM. An equal volume of DM (1 mL) was added to cultures to be included as a DM-only control.

EVs were collected by UF-SEC for MB or MT cultures, and particle counts determined by NTA. Recipient cultures were treated with EVs at the time of switching to DM, with doses ranging from 2×10^2 to 2×10^{11} particles/mL.

Each extracellular protein fraction (nos. 1–15) collected by UF-SEC was collected and protein concentration determined by Micro BCA assay (Thermo Fisher Scientific) according to the manufacturer's instructions. Recipient C2C12 cultures were treated with each fraction (final concentration of 1 μg/mL) at the time of switching to DM.

Statistical analysis

GraphPad Prism 9 software was used for all statistical analyses. Statistical significance was determined by a Student's t test for two sample comparisons, and by one-way ANOVA with Bonferroni post hoc test for comparisons between multiple groups.

DATA AVAILABILITY

All relevant data are included in the manuscript. Raw data are available on request.

SUPPLEMENTAL INFORMATION

Supplemental information can be found online at https://doi.org/10.1016/j.omtn.2023.07.005.

ACKNOWLEDGMENTS

B.H. is supported by a doctoral studentship from the Clarendon Fund. This work was supported by an MRC Programme Grant awarded to M.J.A.W. (MRN0248501).

AUTHOR CONTRIBUTIONS

T.C.R., I.M., P.L.P., S.ELA., and M.J.A.W. conceived the study. B.H., M.C., Y.L., I.M., and T.C.R. performed the experiments and acquired data. T.C.R., P.L.P., and M.J.A.W. supervised the work. B.H. and T.C.R. wrote the first draft of the manuscript. All authors contributed to the final draft of the manuscript.

DECLARATION OF INTERESTS

M.J.A.W. and S.ELA. are founders, shareholders, and consultants for Evox Therapeutics, a biotech company that aims to commercialize extracellular vesicles.

REFERENCES

1. Valadi, H., Ekström, K., Bossios, A., Sjöstrand, M., Lee, J.J., and Lötvall, J.O. (2007). Exosome-mediated transfer of mRNAs and microRNAs is a novel mechanism of genetic exchange between cells. Nat. Cell Biol. *9*, 654–659. https://doi.org/10.1038/ncb1596.
2. Chen, J.-F., Mandel, E.M., Thomson, J.M., Wu, Q., Callis, T.E., Hammond, S.M., Conlon, F.L., and Wang, D.-Z. (2006). The role of microRNA-1 and microRNA-133 in skeletal muscle proliferation and differentiation. Nat. Genet. *38*, 228–233. https://doi.org/10.1038/ng1725.
3. Chen, J.-F., Tao, Y., Li, J., Deng, Z., Yan, Z., Xiao, X., and Wang, D.-Z. (2010). microRNA-1 and microRNA-206 regulate skeletal muscle satellite cell proliferation and differentiation by repressing Pax7. J. Cell Biol. *190*, 867–879. https://doi.org/10.1083/jcb.200911036.
4. Ikeda, S., He, A., Kong, S.W., Lu, J., Bejar, R., Bodyak, N., Lee, K.-H., Ma, Q., Kang, P.M., Golub, T.R., and Pu, W.T. (2009). MicroRNA-1 negatively regulates expression of the hypertrophy-associated calmodulin and Mef2a genes. Mol. Cell Biol. *29*, 2193–2204. https://doi.org/10.1128/MCB.01222-08.
5. Coenen-Stass, A.M.L., Wood, M.J.A., and Roberts, T.C. (2017). Biomarker Potential of Extracellular miRNAs in Duchenne Muscular Dystrophy. Trends Mol. Med. *23*, 989–1001. https://doi.org/10.1016/j.molmed.2017.09.002.
6. Coenen-Stass, A.M.L., Betts, C.A., Lee, Y.F., Mäger, I., Turunen, M.P., El Andaloussi, S., Morgan, J.E., Wood, M.J.A., and Roberts, T.C. (2016). Selective release of muscle-specific, extracellular microRNAs during myogenic differentiation. Hum. Mol. Genet. *25*, 3960–3974. https://doi.org/10.1093/hmg/ddw237.
7. Mizuno, H., Nakamura, A., Aoki, Y., Ito, N., Kishi, S., Yamamoto, K., Sekiguchi, M., Takeda, S., and Hashido, K. (2011). Identification of Muscle-Specific MicroRNAs in Serum of Muscular Dystrophy Animal Models: Promising Novel Blood-Based Markers for Muscular Dystrophy. PLoS One *6*, e18388. https://doi.org/10.1371/journal.pone.0018388.
8. Roberts, T.C., Blomberg, K.E.M., McClorey, G., El Andaloussi, S., Godfrey, C., Betts, C., Coursindel, T., Gait, M.J., Smith, C.I.E., and Wood, M.J.A. (2012). Expression analysis in multiple muscle groups and serum reveals complexity in the microRNA transcriptome of the mdx mouse with implications for therapy. Mol. Ther. Nucleic Acids *1*, e39. https://doi.org/10.1038/mtna.2012.26.
9. Roberts, T.C., Godfrey, C., McClorey, G., Vader, P., Briggs, D., Gardiner, C., Aoki, Y., Sargent, I., Morgan, J.E., and Wood, M.J.A. (2013). Extracellular microRNAs are dynamic non-vesicular biomarkers of muscle turnover. Nucleic Acids Res. *41*, 9500–9513. https://doi.org/10.1093/nar/gkt724.
10. Vignier, N., Amor, F., Fogel, P., Duvallet, A., Poupiot, J., Charrier, S., Arock, M., Montus, M., Nelson, I., Richard, I., et al. (2013). Distinctive Serum miRNA Profile in Mouse Models of Striated Muscular Pathologies. PLoS One *8*, e55281. https://doi.org/10.1371/journal.pone.0055281.
11. Goyenvalle, A., Babbs, A., Wright, J., Wilkins, V., Powell, D., Garcia, L., and Davies, K.E. (2012). Rescue of severely affected dystrophin/utrophin-deficient mice through scAAV-U7snRNA-mediated exon skipping. Hum. Mol. Genet. *21*, 2559–2571. https://doi.org/10.1093/hmg/dds082.
12. Jeanson-Leh, L., Lameth, J., Krimi, S., Buisset, J., Amor, F., Le Guiner, C., Barthélémy, I., Servais, L., Blot, S., Voit, T., and Israeli, D. (2014). Serum Profiling Identifies Novel Muscle miRNA and Cardiomyopathy-Related miRNA Biomarkers in Golden Retriever Muscular Dystrophy Dogs and Duchenne Muscular Dystrophy Patients. Am. J. Pathol. *184*, 2885–2898. https://doi.org/10.1016/j.ajpath.2014.07.021.
13. Duguez, S., Duddy, W., Johnston, H., Lainé, J., Le Bihan, M.C., Brown, K.J., Bigot, A., Hathout, Y., Butler-Browne, G., and Partridge, T. (2013). Dystrophin deficiency leads to disturbance of LAMP1-vesicle-associated protein secretion. Cell. Mol. Life Sci. *70*, 2159–2174. https://doi.org/10.1007/s00018-012-1248-2.
14. Coenen-Stass, A.M.L., Pauwels, M.J., Hanson, B., Martin Perez, C., Conceição, M., Wood, M.J.A., Mäger, I., and Roberts, T.C. (2019). Extracellular microRNAs exhibit sequence-dependent stability and cellular release kinetics. RNA Biol. *16*, 696–706. https://doi.org/10.1080/15476286.2019.1582956.
15. Forterre, A., Jalabert, A., Berger, E., Baudet, M., Chikh, K., Errazuriz, E., De Larichaudy, J., Chanon, S., Weiss-Gayet, M., Hesse, A.-M., et al. (2014). Proteomic analysis of C2C12 myoblast and myotube exosome-like vesicles: a new paradigm for myoblast-myotube cross talk? PLoS One *9*, e84153. https://doi.org/10.1371/journal.pone.0084153.
16. Guescini, M., Guidolin, D., Vallorani, L., Casadei, L., Gioacchini, A.M., Tibollo, P., Battistelli, M., Falcieri, E., Battistin, L., Agnati, L.F., and Stocchi, V. (2010). C2C12 myoblasts release micro-vesicles containing mtDNA and proteins involved in signal transduction. Exp. Cell Res. *316*, 1977–1984. https://doi.org/10.1016/j.yexcr.2010.04.006.
17. Madison, R.D., McGee, C., Rawson, R., and Robinson, G.A. (2014). Extracellular vesicles from a muscle cell line (C2C12) enhance cell survival and neurite outgrowth of a motor neuron cell line (NSC-34). J. Extracell. Vesicles *3*, 22865. https://doi.org/10.3402/jev.v3.22865.
18. Fry, C.S., Kirby, T.J., Kosmac, K., McCarthy, J.J., and Peterson, C.A. (2017). Myogenic Progenitor Cells Control Extracellular Matrix Production by Fibroblasts during Skeletal Muscle Hypertrophy. Cell Stem Cell *20*, 56–69. https://doi.org/10.1016/j.stem.2016.09.010.
19. Choi, J.S., Yoon, H.I., Lee, K.S., Choi, Y.C., Yang, S.H., Kim, I.-S., and Cho, Y.W. (2016). Exosomes from differentiating human skeletal muscle cells trigger myogenesis of stem cells and provide biochemical cues for skeletal muscle regeneration. J. Control. Release *222*, 107–115. https://doi.org/10.1016/j.jconrel.2015.12.018.
20. Nakamura, Y., Miyaki, S., Ishitobi, H., Matsuyama, S., Nakasa, T., Kamei, N., Akimoto, T., Higashi, Y., and Ochi, M. (2015). Mesenchymal-stem-cell-derived exosomes accelerate skeletal muscle regeneration. FEBS Lett. *589*, 1257–1265. https://doi.org/10.1016/j.febslet.2015.03.031.
21. Fry, C.S., Lee, J.D., Jackson, J.R., Kirby, T.J., Stasko, S.A., Liu, H., Dupont-Versteegden, E.E., McCarthy, J.J., and Peterson, C.A. (2014). Regulation of the muscle fiber micro environment by activated satellite cells during hypertrophy. FASEB J. *28*, 1654–1665. https://doi.org/10.1096/fj.13-239426.

22. Wagner, J., Riwanto, M., Besler, C., Knau, A., Fichtlscherer, S., Röxe, T., Zeiher, A.M., Landmesser, U., and Dimmeler, S. (2013). Characterization of levels and cellular transfer of circulating lipoprotein-bound microRNAs. Arterioscler. Thromb. Vasc. Biol. *33*, 1392–1400. https://doi.org/10.1161/ATVBAHA.112.300741.

23. Chan, C.Y.X., Masui, O., Krakovska, O., Belozerov, V.E., Voisin, S., Ghanny, S., Chen, J., Moyez, D., Zhu, P., Evans, K.R., et al. (2011). Identification of differentially regulated secretome components during skeletal myogenesis. Mol. Cell. Proteomics *10*. M110.004804. https://doi.org/10.1074/mcp.M110.004804.

24. Henningsen, J., Rigbolt, K.T.G., Blagoev, B., Pedersen, B.K., and Kratchmarova, I. (2010). Dynamics of the skeletal muscle secretome during myoblast differentiation. Mol. Cell. Proteomics *9*, 2482–2496. https://doi.org/10.1074/mcp.M110.002113.

25. Kosaka, N., Iguchi, H., Yoshioka, Y., Takeshita, F., Matsuki, Y., and Ochiya, T. (2010). Secretory Mechanisms and Intercellular Transfer of MicroRNAs in Living Cells. J. Biol. Chem. *285*, 17442–17452. https://doi.org/10.1074/jbc.M110.107821.

26. Maguire, C.A., Balaj, L., Sivaraman, S., Crommentuijn, M.H.W., Ericsson, M., Mincheva-Nilsson, L., Baranov, V., Gianni, D., Tannous, B.A., Sena-Esteves, M., et al. (2012). Microvesicle-associated AAV Vector as a Novel Gene Delivery System. Mol. Ther. *20*, 960–971. https://doi.org/10.1038/mt.2011.303.

27. Ostrowski, M., Carmo, N.B., Krumeich, S., Fanget, I., Raposo, G., Savina, A., Moita, C.F., Schauer, K., Hume, A.N., Freitas, R.P., et al. (2010). Rab27a and Rab27b control different steps of the exosome secretion pathway. Nat. Cell Biol. *12*, 19–30. https://doi.org/10.1038/ncb2000.

28. Nordin, J.Z., Lee, Y., Vader, P., Mäger, I., Johansson, H.J., Heusermann, W., Wiklander, O.P.B., Hällbrink, M., Seow, Y., Bultema, J.J., et al. (2015). Ultrafiltration with size-exclusion liquid chromatography for high yield isolation of extracellular vesicles preserving intact biophysical and functional properties. Nanomedicine. *11*, 879–883. https://doi.org/10.1016/j.nano.2015.01.003.

29. Thermo Fisher Scientific Opti-MEM^{TM} I Reduced Serum Medium Product Information Sheet.

30. Okabe, S., Forsberg-Nilsson, K., Spiro, A.C., Segal, M., and McKay, R.D. (1996). Development of neuronal precursor cells and functional postmitotic neurons from embryonic stem cells in vitro. Mech. Dev. *59*, 89–102. https://doi.org/10.1016/0925-4773(96)00572-2.

31. Chal, J., Al Tanoury, Z., Hestin, M., Gobert, B., Aivio, S., Hick, A., Cherrier, T., Nesmith, A.P., Parker, K.K., and Pourquié, O. (2016). Generation of human muscle fibers and satellite-like cells from human pluripotent stem cells in vitro. Nat. Protoc. *11*, 1833–1850. https://doi.org/10.1038/nprot.2016.110.

32. Swartz, E.W., Baek, J., Pribadi, M., Wojta, K.J., Almeida, S., Karydas, A., Gao, F.-B., Miller, B.L., and Coppola, G. (2016). A Novel Protocol for Directed Differentiation of C9orf72-Associated Human Induced Pluripotent Stem Cells Into Contractile Skeletal Myotubes. Stem Cells Transl. Med. *5*, 1461–1472. https://doi.org/10.5966/sctm.2015-0340.

33. Trajkovic, K., Hsu, C., Chiantia, S., Rajendran, L., Wenzel, D., Wieland, F., Schwille, P., Brügger, B., and Simons, M. (2008). Ceramide triggers budding of exosome vesicles into multivesicular endosomes. Science *319*, 1244–1247. https://doi.org/10.1126/science.1153124.

34. Stenmark, H. (2009). Rab GTPases as coordinators of vesicle traffic. Nat. Rev. Mol. Cell Biol. *10*, 513–525. https://doi.org/10.1038/nrm2728.

35. Fukuda, M. (2008). Regulation of secretory vesicle traffic by Rab small GTPases. Cell. Mol. Life Sci. *65*, 2801–2813. https://doi.org/10.1007/s00018-008-8351-4.

36. Bobrie, A., Krumeich, S., Reyal, F., Recchi, C., Moita, L.F., Seabra, M.C., Ostrowski, M., and Théry, C. (2012). Rab27a Supports Exosome-Dependent and -Independent Mechanisms That Modify the Tumor Microenvironment and Can Promote Tumor Progression. Cancer Res. *72*, 4920–4930. https://doi.org/10.1158/0008-5472.CAN-12-0925.

37. Bost, J.P., Saher, O., Hagey, D., Mamand, D.R., Liang, X., Zheng, W., Corso, G., Gustafsson, O., Görgens, A., Smith, C.E., et al. (2022). Growth Media Conditions Influence the Secretion Route and Release Levels of Engineered Extracellular Vesicles. Adv. Healthc. Mater. *11*, e2101658. https://doi.org/10.1002/adhm.202101658.

38. Deng, Z.b., Poliakov, A., Hardy, R.W., Clements, R., Liu, C., Liu, Y., Wang, J., Xiang, X., Zhang, S., Zhuang, X., et al. (2009). Adipose tissue exosome-like vesicles mediate activation of macrophage-induced insulin resistance. Diabetes *58*, 2498–2505. https://doi.org/10.2337/db09-0216.

39. Mytidou, C., Koutsoulidou, A., Katsioloudi, A., Prokopi, M., Kapnisis, K., Michailidou, K., Anayiotos, A., and Phylactou, L.A. (2021). Muscle-derived exosomes encapsulate myomiRs and are involved in local skeletal muscle tissue communication. FASEB J. *35*, e21279. https://doi.org/10.1096/fj.201902468RR.

40. Forterre, A., Jalabert, A., Chikh, K., Pesenti, S., Euthine, V., Granjon, A., Errazuriz, E., Lefai, E., Vidal, H., and Rome, S. (2014). Myotube-derived exosomal miRNAs downregulate Sirtuin1 in myoblasts during muscle cell differentiation. Cell Cycle *13*, 78–89. https://doi.org/10.4161/cc.26808.

41. Théry, C., Witwer, K.W., Aikawa, E., Alcaraz, M.J., Anderson, J.D., Andriantsitohaina, R., Antoniou, A., Arab, T., Archer, F., Atkin-Smith, G.K., et al. (2018). Minimal information for studies of extracellular vesicles 2018 (MISEV2018): a position statement of the International Society for Extracellular Vesicles and update of the MISEV2014 guidelines. J. Extracell. Vesicles *7*, 1535750. https://doi.org/10.1080/20013078.2018.1535750.

42. Cvjetkovic, A., Lötvall, J., and Lässer, C. (2014). The influence of rotor type and centrifugation time on the yield and purity of extracellular vesicles. J. Extracell. Vesicles *3*, 23111. https://doi.org/10.3402/jev.v3.23111.

43. Van Deun, J., Mestdagh, P., Sormunen, R., Cocquyt, V., Vermaelen, K., Vandesompele, J., Bracke, M., De Wever, O., and Hendrix, A. (2014). The impact of disparate isolation methods for extracellular vesicles on downstream RNA profiling. J. Extracell. Vesicles *3*, 24858. https://doi.org/10.3402/jev.v3.24858.

44. Chevillet, J.R., Kang, Q., Ruf, I.K., Briggs, H.A., Vojtech, L.N., Hughes, S.M., Cheng, H.H., Arroyo, J.D., Meredith, E.K., Gallichotte, E.N., et al. (2014). Quantitative and stoichiometric analysis of the microRNA content of exosomes. Proc. Natl. Acad. Sci. USA *111*, 14888–14893. https://doi.org/10.1073/pnas.1408301111.

45. Albanese, M., Chen, Y.-F.A., Hüls, C., Gärtner, K., Tagawa, T., Mejias-Perez, E., Keppler, O.T., Göbel, C., Zeidler, R., Shein, M., et al. (2021). MicroRNAs are minor constituents of extracellular vesicles that are rarely delivered to target cells. PLoS Genet. *17*, e1009951. https://doi.org/10.1371/journal.pgen.1009951.

46. Lötvall, J., Hill, A.F., Hochberg, F., Buzás, E.I., Di Vizio, D., Gardiner, C., Gho, Y.S., Kurochkin, I.V., Mathivanan, S., Quesenberry, P., et al. (2014). Minimal experimental requirements for definition of extracellular vesicles and their functions: a position statement from the International Society for Extracellular Vesicles. J. Extracell. Vesicles *3*, 26913. https://doi.org/10.3402/jev.v3.26913.

47. Karttunen, J., Heiskanen, M., Navarro-Ferrandis, V., Das Gupta, S., Lipponen, A., Puhakka, N., Rilla, K., Koistinen, A., and Pitkänen, A. (2019). Precipitation-based extracellular vesicle isolation from rat plasma co-precipitate vesicle-free microRNAs. J. Extracell. Vesicles *8*, 1555410. https://doi.org/10.1080/20013078.2018.1555410.

48. Whittaker, T.E., Nagelkerke, A., Nele, V., Kauscher, U., and Stevens, M.M. (2020). Experimental artefacts can lead to misattribution of bioactivity from soluble mesenchymal stem cell paracrine factors to extracellular vesicles. J. Extracell. Vesicles *9*, 1807674. https://doi.org/10.1080/20013078.2020.1807674.

49. Tóth, E.Á., Turiák, L., Visnovitz, T., Cserép, C., Mázló, A., Sódar, B.W., Försönits, A.I., Petővári, G., Sebestyén, A., Komlósi, Z., et al. (2021). Formation of a protein corona on the surface of extracellular vesicles in blood plasma. J. Extracell. Vesicles *10*, e12140. https://doi.org/10.1002/jev2.12140.

50. Ahmad, S.S., Ahmad, K., Lee, E.J., Lee, Y.-H., and Choi, I. (2020). Implications of Insulin-Like Growth Factor-1 in Skeletal Muscle and Various Diseases. Cells *9*. https://doi.org/10.3390/cells9081773.

51. Le Roith, D. (1997). Insulin-Like Growth Factors. N. Engl. J. Med. *336*, 633–640. https://doi.org/10.1056/NEJM199702273360907.

52. Taniguchi, C.M., Emanuelli, B., and Kahn, C.R. (2006). Critical nodes in signalling pathways: insights into insulin action. Nat. Rev. Mol. Cell Biol. *7*, 85–96. https://doi.org/10.1038/nrm1837.

53. Conejo, R., and Lorenzo, M. (2001). Insulin signaling leading to proliferation, survival, and membrane ruffling in C2C12 myoblasts. J. Cell. Physiol. *187*, 96–108. https://doi.org/10.1002/1097-4652(2001)9999:9999<::AID-JCP1058>3.0.CO;2-V.

54. Conejo, R., Valverde, A.M., Benito, M., and Lorenzo, M. (2001). Insulin produces myogenesis in C2C12 myoblasts by induction of NF-κB and downregulation of

AP-1 activities. J. Cell. Physiol. *186*, 82–94. https://doi.org/10.1002/1097-4652(200101)186:1<82::AID-JCP1001>3.0.CO;2-R.

55. Liu, D., Moberg, E., Kollind, M., Lins, P.E., and Adamson, U. (1991). A high concentration of circulating insulin suppresses the glucagon response to hypoglycemia in normal man. J. Clin. Endocrinol. Metab. *73*, 1123–1128. https://doi.org/10.1210/jcem-73-5-1123.

56. Hu, W., Ru, Z., Zhou, Y., Xiao, W., Sun, R., Zhang, S., Gao, Y., Li, X., Zhang, X., and Yang, H. (2019). Lung cancer-derived extracellular vesicles induced myotube atrophy and adipocyte lipolysis via the extracellular IL-6-mediated STAT3 pathway. Biochim. Biophys. Acta, Mol. Cell Biol. Lipids *1864*, 1091–1102. https://doi.org/10.1016/j.bbalip.2019.04.006.

57. Hettinger, Z.R., Kargl, C.K., Shannahan, J.H., Kuang, S., and Gavin, T.P. (2021). Extracellular vesicles released from stress-induced prematurely senescent myoblasts impair endothelial function and proliferation. Exp. Physiol. *106*, 2083–2095. https://doi.org/10.1113/EP089423.

58. Childs, B.G., Gluscevic, M., Baker, D.J., Laberge, R.-M., Marquess, D., Dananberg, J., and van Deursen, J.M. (2017). Senescent cells: an emerging target for diseases of ageing. Nat. Rev. Drug Discov. *16*, 718–735. https://doi.org/10.1038/nrd.2017.116.

59. Roberts, T.C., Etxaniz, U., Dall'Agnese, A., Wu, S.-Y., Chiang, C.-M., Brennan, P.E., Wood, M.J.A., and Puri, P.L. (2017). BRD3 and BRD4 BET Bromodomain Proteins Differentially Regulate Skeletal Myogenesis. Sci. Rep. *7*, 6153. https://doi.org/10.1038/s41598-017-06483-7.

60. Bustin, S.A., Benes, V., Garson, J.A., Hellemans, J., Huggett, J., Kubista, M., Mueller, R., Nolan, T., Pfaffl, M.W., Shipley, G.L., et al. (2009). The MIQE Guidelines: Minimum Information for Publication of Quantitative Real-Time PCR Experiments. Clin. Chem. *55*, 611–622. https://doi.org/10.1373/clinchem.2008.112797.

61. Pfaffl, M.W. (2001). A new mathematical model for relative quantification in real-time RT-PCR. Nucleic Acids Res. *29*, e45. https://doi.org/10.1093/nar/29.9.e45.

62. Roberts, T.C., Coenen-Stass, A.M.L., Betts, C.A., and Wood, M.J.A. (2014). Detection and quantification of extracellular microRNAs in murine biofluids. Biol. Proced. Online *16*, 5. https://doi.org/10.1186/1480-9222-16-5.

63. Roberts, T.C., Coenen-Stass, A.M.L., and Wood, M.J.A. (2014). Assessment of RT-qPCR normalization strategies for accurate quantification of extracellular microRNAs in murine serum. PLoS One *9*, e89237. https://doi.org/10.1371/journal.pone.0089237.

64. Coenen-Stass, A.M.L., Sork, H., Gatto, S., Godfrey, C., Bhomra, A., Krjutškov, K., Hart, J.R., Westholm, J.O., O'Donovan, L., Roos, A., et al. (2018). Comprehensive RNA-Sequencing Analysis in Serum and Muscle Reveals Novel Small RNA Signatures with Biomarker Potential for DMD. Mol. Ther. Nucleic Acids *13*, 1–15. https://doi.org/10.1016/j.omtn.2018.08.005.

65. Chwalenia, K., Oieni, J., Zemła, J., Lekka, M., Ahlskog, N., Coenen-Stass, A.M.L., McClorey, G., Wood, M.J.A., Lomonosova, Y., and Roberts, T.C. (2022). PPMO-Mediated Exon Skipping Induces Uniform Sarcolemmal Dystrophin Rescue with Dose-dependent Restoration of Circulating microRNA Biomarkers and Muscle Biophysical Properties. https://doi.org/10.1101/2022.01.25.477672.

OMTN, Volume *33*

Supplemental information

EV-mediated promotion of myogenic differentiation is dependent on dose, collection medium, and isolation method

Britt Hanson, Ioulia Vorobieva, Wenyi Zheng, Mariana Conceição, Yulia Lomonosova, Imre Mäger, Pier Lorenzo Puri, Samir El Andaloussi, Matthew J.A. Wood, and Thomas C. Roberts

Supplemental Information

Table S1

Target sequences for SMARTpool siRNAs used in this study.

ID	Target sequence (5' to 3')
siRab27a	CGGAUGGAGAUUACGAUUA
	CAGGAGAGGUUUCGUAGCU
	GUACAGAGCCAAUGGGCCA
	GGGCAUUGAUUUCAGGGAA
siRab27b	GCAGAUUAGAAGCUAGUAU
	GGGAAUAGAUUUUCGGGAA
	GGAAGUCAAUGAACGGCAA
	GCAGAGUAGUCAUAGUGUU
siCtrl	UGGUUUACAUGUCGACUAA
	UGGUUUACAUGUUGUGUGA
	UGGUUUACAUGUUUUCUGA
	UGGUUUACAUGUUUUCCUA

Table S2

Antibodies used in this study.

MF 20 primary monoclonal antibody, developed by Fischman, D.A. at Weill Cornell Medical College, was obtained from the Developmental Studies Hybridoma Bank, created by the NICHD of the NIH and maintained at The University of Iowa, Department of Biology, Iowa City, IA 52242, USA. WB, western blot, IF, immunofluorescence.

Target protein	Host	Product ID	Manufacturer	Dilution
**Western Blot**				
**Primary Abs**				
Anti-PDCD6IP (Alix)	Mouse	ab117600	Abcam	1:1,000
Anti-CANX (Calnexin)	Rabbit	ab22595	Abcam	1:1,000
Anti-CD9	Rabbit	ab92726	Abcam	1:2,000
**Secondary Abs**				
Anti-mouse IgG, HRP-linked	Horse	7076	Cell Signaling Technology	1:5,000
**Immunofluorescence**				
**Primary Abs**				
Anti-MHC	Mouse	MF 20	Abcam	1:20
**Secondary Abs**				
Anti-rat IgG Alexa Fluor 488	Goat	ab150157	Abcam	1:500

Table S3

RT-qPCR primer sequences used in this study.

ID	Sequence (5' to 3')
**qRab27a-Fwd**	CGACCTGACAAATGAGCAAAG
**qRab27a-Rev**	CCTCTTTCACTGCCCTCTG
**qRab27b-Fwd**	CAGACCTGCCAGACCAAAG
**qRab27b-Rev**	AGCGTTTCCACTGACTTCTC
**qRplp0-Fwd**	AAGCAAAGGAAGAGTCGGAG
**qRplp0-Rev**	CCAGACCGGAGTTTTAAGAGAAG

Table S4

miRNA small RNA TaqMan RT-qPCR assays used in this study.

All products were purchased from Thermo Fisher Scientific.

Target	Product ID	Detection Channel
**mmu-miR-1a-3p**	002222	FAM
**mmu-miR-133a-3p**	002246	FAM
**mmu-miR-206-3p**	000510	FAM
**cel-miR-39**	000200	FAM

Table S5

List of oligonucleotides used in this study.

Oligonucleotides were purchased from IDT (Leuven, Belgium).

ID	Sequence (5' to 3')
mmu-miR-1a-3p	UGGAAUGUAAAGAAGUAUGUAU
mmu-miR-133a-3p	UUUGGUCCCCUUCAACCAGCUG
mmu-miR-206-3p	UGGAAUGUAAGGAAGUGUGUGG
cel-miR-39	UCACCGGGUGUAAAUCAGCUUG

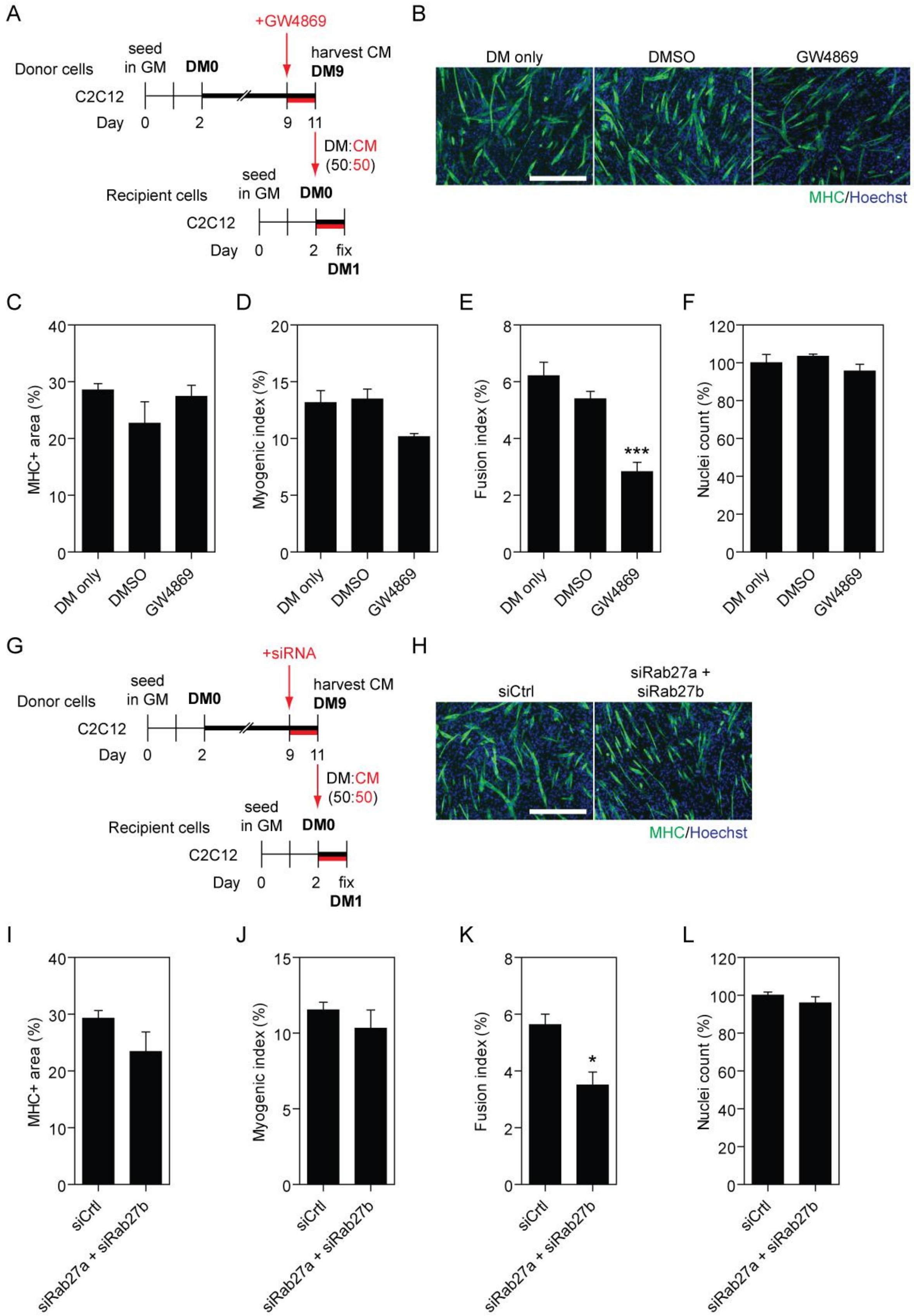

A
+GW4869
seed in GM
Donor cells
DM0
harvest CM
DM9
C2C12
Day 0 2 9 11
DM:CM (50:50)
seed in GM
Recipient cells
DM0
C2C12
Day 0 2 fix
DM1
B
DM only
DMSO
GW4869
MHC/Hoechst
C
MHC+ area (%)
D
Myogenic index (%)
E
Fusion index (%)
***
F
Nuclei count (%)
DM only
DMSO
GW4869
G
+siRNA
H
siCtrl
siRab27a + siRab27b
I
J
K
*
L
siCrtl
siRab27a + siRab27b

Figure S1

Treatment of donor cultures with exosome biogenesis inhibitors reduces myogenic differentiation in recipient cultures following conditioned media transfer.

(**A**) Donor C2C12 cells were cultured in GM for two days and then switched to DM for seven days. Cultures were treated with 10 µM GW4869 and conditioned media (CM) collected 2 days later. Untreated (DM only) and DMSO treated cultures served as controls. Recipient cultures were treated with a 50:50 mixture of CM and fresh differentiation media (DM) at the point of initiating differentiation. Cells were fixed after 24 hours and myogenic differentiation was assessed in the recipient cultures by (**B**) MHC IF, and quantified by (**C**) measuring the MHC+ area, and by calculating the (**D**) myogenic and (**E**) fusion indices. (**F**) The total number of nuclei per representative field of view are shown as a percentage relative to the control group. (**G**) Donor C2C12 cells were cultured as above, and treated with a 50:50 mixture of siRNAs targeting the exosome biogenesis factors *Rab27a* and *Rab27b* (final concentration 100 nM) at differentiation day 7. Conditioned media was harvested 2 days later. A non-targeting siRNA (siCtrl) served as a control). Recipient cultures were treated with a 50:50 mixture of CM and fresh differentiation media (DM) at the point of initiating differentiation. Cells were fixed after 24 hours and myogenic differentiation was assessed in the recipient cultures by (**H**) MHC IF, and quantified by (**I**) measuring the MHC+ area, and by calculating the (**J**) myogenic and (K) fusion indices. (**L**) The total number of nuclei per representative field of view are shown as a percentage relative to the control group. All microscopy images were taken at 10× magnification. Scale bar represents 400 µm. Values are mean + SEM (n=4). Statistical significance was determined by one-way ANOVA with Bonferroni *post hoc* test or Student's t-test, as appropriate, ***P<0.001, *P<0.05.

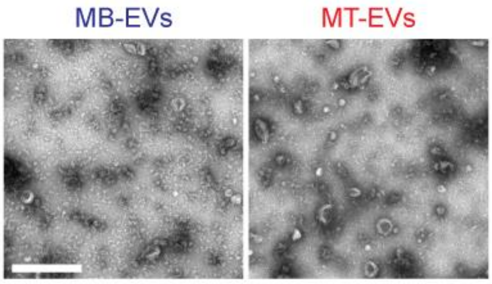

Figure S2

Analysis of EV preparations by transmission electron microscopy.

EVs were collected from myoblasts (MBs) or myotubes (MTs) as described in **Figure 3A** and preparations were analysed by transmission electron microscopy. Vesicles with ‘cup-shaped’ morphology characteristic of exosomes are observed. Scale bars represent 500 nm.

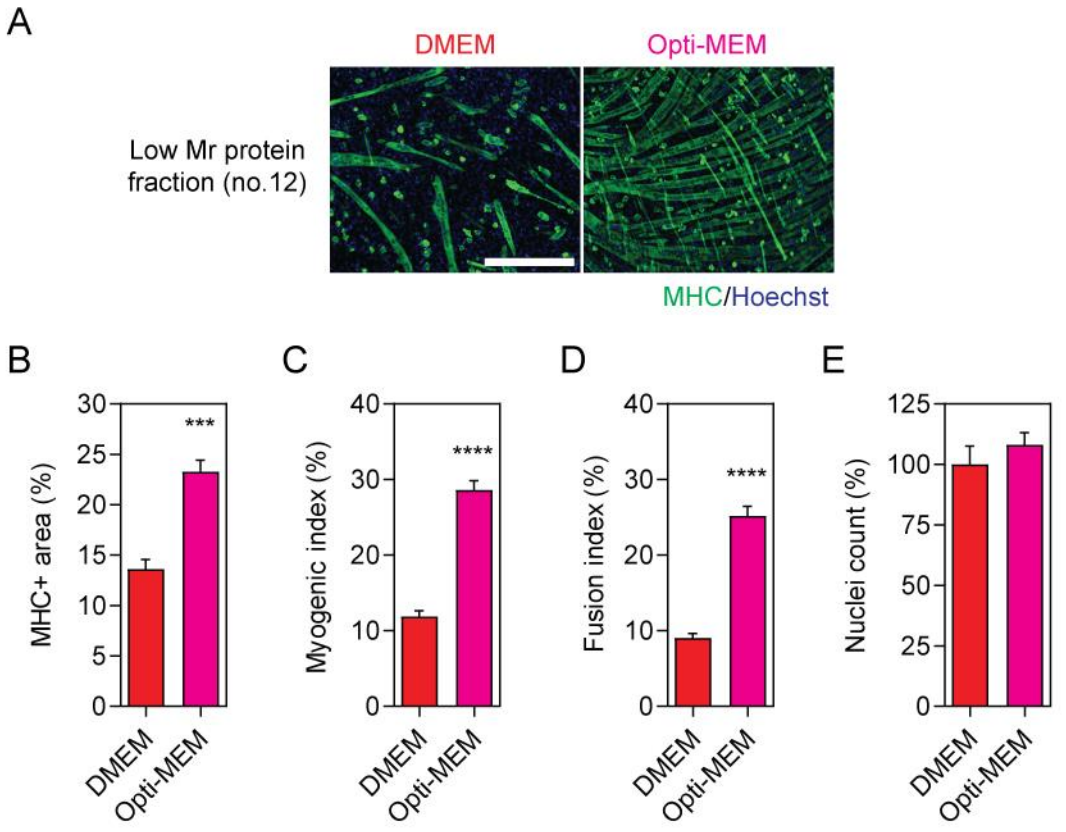

Figure S3

Low molecular weight protein obtained from unconditioned Opti-MEM media is highly pro-myogenic.

Fresh Opti-MEM and DMEM were fractionated by UF-SEC and protein fraction no. 12 collected as shown in **Figure 5B**. Recipient C2C12 cultures were treated with 1 µg/ml of purified protein fraction at the time of switching to DM. (**A**) Myogenic differentiation was assessed by MHC IF at DM2 and quantified by measuring (**B**) MHC+ area, (**C**) myogenic index, and (**D**) fusion index. (**E**) The total number of nuclei per representative field of view are shown as a percentage relative to the control group. All microscopy images were taken at 10× magnification. Scale bar represents 400 µm. All values are mean + SEM (*n*=4). Statistical significance was determined by a Student's *t* test, ***P<0.001, ****P<0.0001.

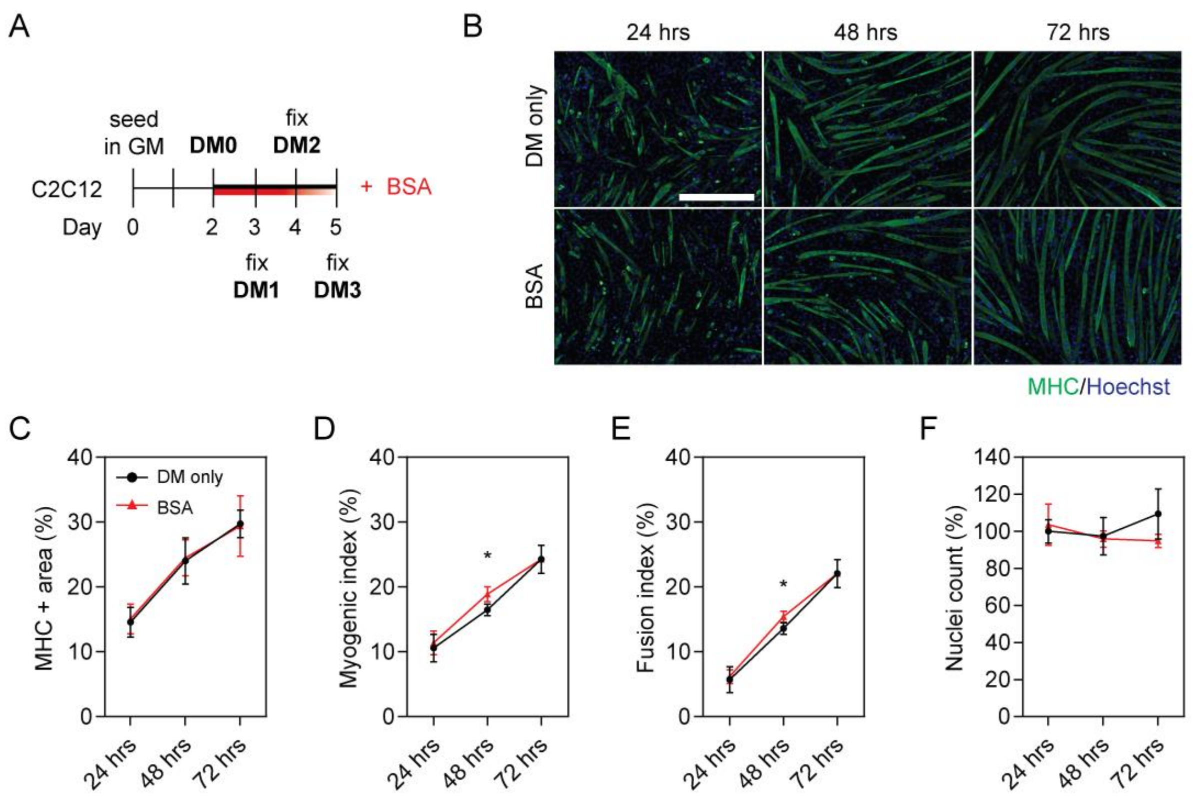

Figure S4

A non-specific increase in extracellular protein does not enhance myogenic differentiation.

(**A**) C2C12 myoblasts were grown in GM for two days, and treated with 5 µg/ml of bovine serum albumin (BSA) at the time of switching to DM. (**B**) Myogenic differentiation was assessed by MHC IF at DM1, DM2 and DM3, and quantified by measuring (**C**) MHC+ area, (**D**) myogenic index, and (**E**) fusion index. Untreated (DM only) cultures were included as negative controls. (**F**) The total number of nuclei per representative field of view are shown as a percentage relative to the control group. All microscopy images were taken at 10× magnification. Scale bar represents 400 µm. All values are mean ± SEM (*n*=4). Statistical significance was determined by Student's *t* test, *$P<0.05$.

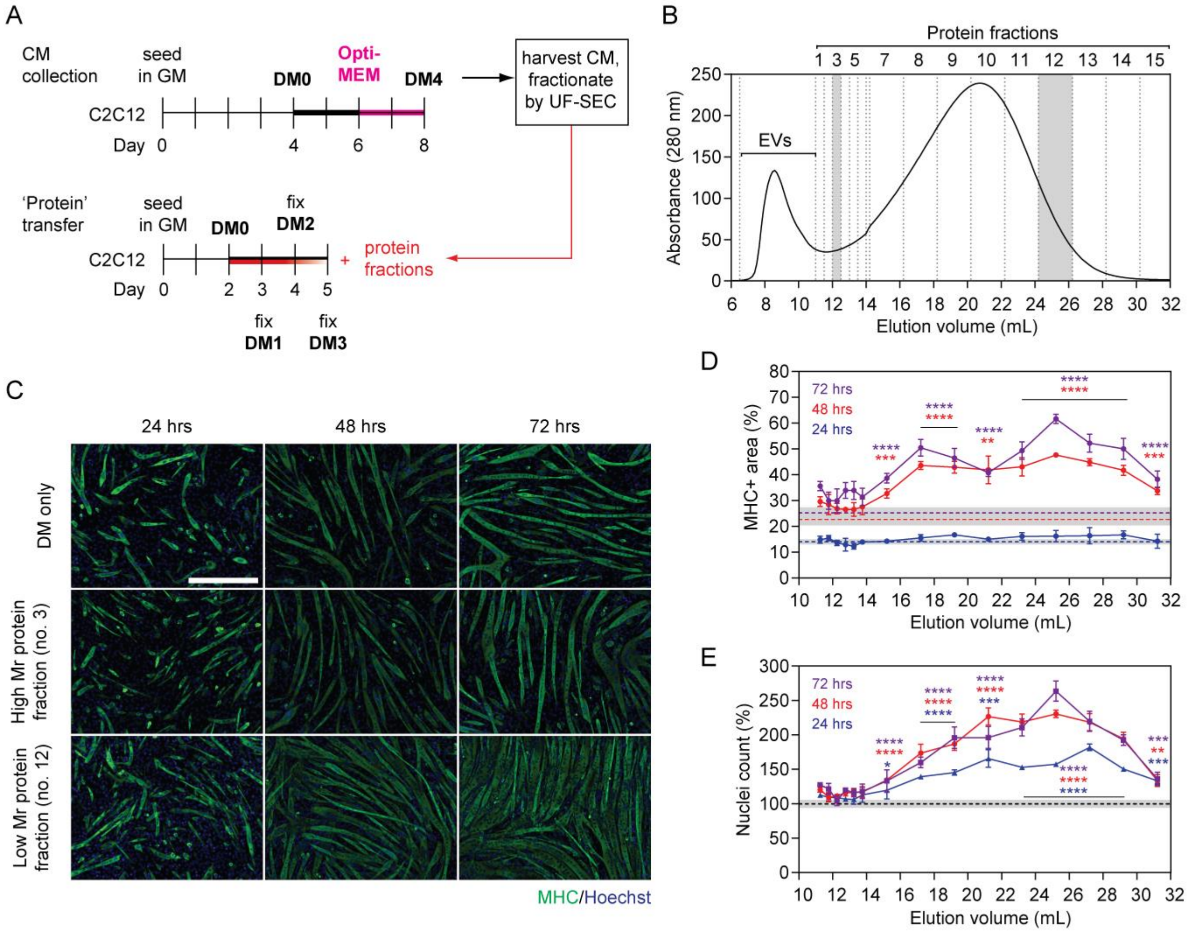

A
CM collection
seed in GM
DM0
Opti-MEM
DM4
C2C12
Day 0 4 6 8
harvest CM, fractionate by UF-SEC
'Protein' transfer
seed in GM
DM0
fix DM2
C2C12
Day 0 2 3 4 5
+ protein fractions
fix DM1
fix DM3
B
Protein fractions
1 3 5 7 8 9 10 11 12 13 14 15
EVs
Absorbance (280 nm)
Elution volume (mL)
C
24 hrs
48 hrs
72 hrs
DM only
High Mr protein fraction (no. 3)
Low Mr protein fraction (no. 12)
MHC/Hoechst
D
72 hrs
48 hrs
24 hrs
MHC+ area (%)
Elution volume (mL)
E
72 hrs
48 hrs
24 hrs
Nuclei count (%)
Elution volume (mL)

Figure S5

Low molecular weight myotube-derived secreted protein enhances myogenic differentiation when purified from Opti-MEM collection media.

(**A**) C2C12 myotube-derived secreted protein fractions were isolated by UF-SEC from CM collected in Opti-MEM. Recipient cultures were treated with 1 μg/ml of purified extracellular protein from each fraction at the time of switching to DM. (**B**) The non-vesicular-associated protein eluates from the SEC column were measured by UV spectrophotometry absorbance at 280 nm. Two fractions (no. 3 and no. 12) were selected to represent high and low Mr extracellular protein fractions, respectively (indicated by grey shading). (**C**) Myogenic differentiation in recipient cultures was assessed by MHC IF at DM1 (24 hours), DM2 (48 hours), and DM3 (72 hours). Representative images from the high and low Mr extracellular protein fractions are shown from each time point. (**D**) Myogenic differentiation was quantified by measuring the MHC+ area. The means of corresponding DM only control groups from each time point are shown ± SEM (grey shaded area). (**E**) The total number of nuclei per representative field of view are shown as a percentage relative to the control group (data were scaled such that the mean of the control group was returned to a value of 100%). Untreated (DM only) cultures were included as negative controls. All microscopy images were taken at 10× magnification. Scale bar represents 400 μm. Values are mean ± SEM (*n*=4). Statistical significance was determined by a Student's *t*-test, $^{**}P<0.01$, $^{***}P<0.001$, and $^{****}P<0.0001$ (for ease of interpretation test results are only shown for fraction nos. 7-15). The colour of the significance indicators corresponds to the respective comparison relative to the DM only control at each time point (i.e. blue for the 24 hour time point, red for 48 hours, and purple for 72 hours).

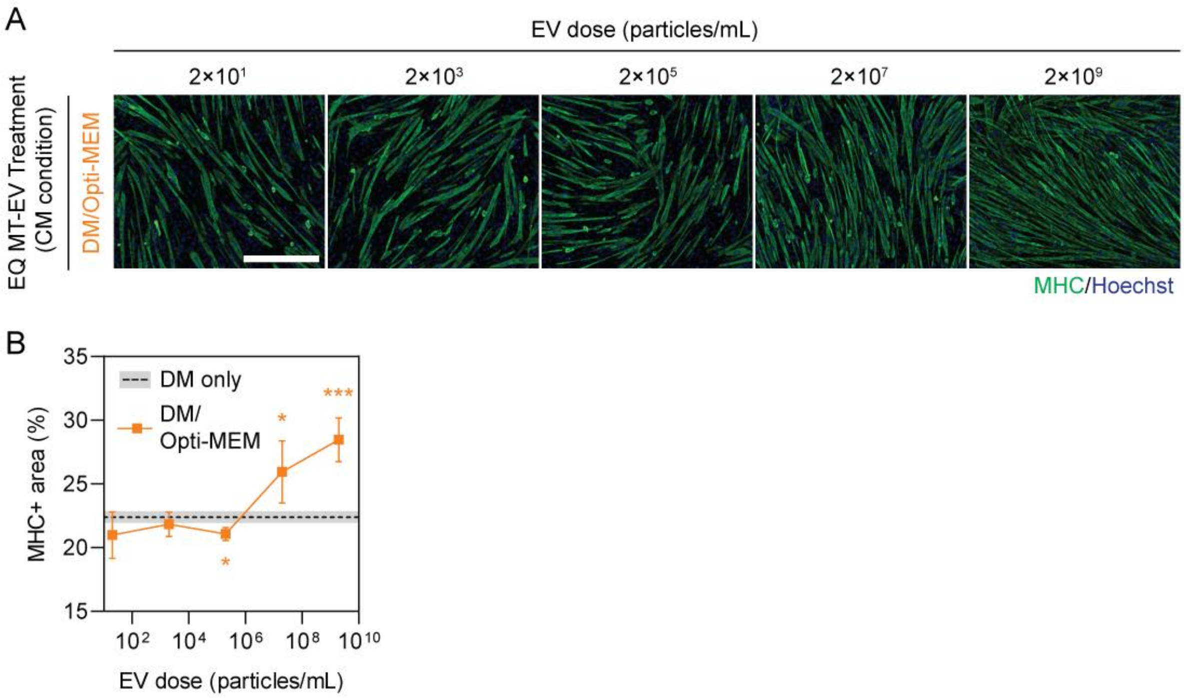
A
EV dose (particles/mL)
2×10¹
2×10³
2×10⁵
2×10⁷
2×10⁹
EQ MT-EV Treatment (CM condition)
DM/Opti-MEM
MHC/Hoechst
B
DM only
DM/ Opti-MEM
MHC+ area (%)
EV dose (particles/mL)

Figure S6

EQ MT-EVs obtained from Opti-MEM collection media exhibit pro-myogenic effects at high doses.

EVs were isolated by ExoQuick polymer precipitation (EQ MT-EVs) from C2C12 myotubes and collected in Opti-MEM isolation media. Myoblasts were grown in GM for four days, DM for two days, and isolation medium (Opti-MEM) for a further two days. Recipient myoblast cultures were treated with a range of EQ MT-EV doses. (**A**) Myogenic differentiation was assessed by MHC IF at DM2 and quantified by (**B**) measuring the MHC+ area. All microscopy images were taken at 10× magnification. Scale bar represents 400 µm. All values are mean ± SEM (n=4). Untreated (DM only) cultures were included as negative controls. Statistical significance was determined by Student's t-test, *P<0.05, ***P<0.001.
